# Meta-analysis of genome-wide expression patterns associated with behavioral maturation in honey bees

**DOI:** 10.1186/1471-2164-9-503

**Published:** 2008-10-24

**Authors:** Heather A Adams, Bruce R Southey, Gene E Robinson, Sandra L Rodriguez-Zas

**Affiliations:** 1 Department of Animal Sciences, University of Illinois, Urbana, Illinois 61801, USA; 2 Institute for Genomic Biology, University of Illinois, Urbana, Illinois 61801, USA; 3 Department of Chemistry, University of Illinois, Urbana, Illinois 61801, USA; 4 Department of Computer Science, University of Illinois, Urbana, Illinois 61801, USA; 5 Neuroscience Program, University of Illinois, Urbana, Illinois 61801, USA; 6 Department of Entomology, University of Illinois, Urbana, Illinois 61801, USA; 7 Department of Statistics, University of Illinois, Urbana, Illinois 61801, USA

## Abstract

**Background:**

The information from multiple microarray experiments can be integrated in an objective manner *via *meta-analysis. However, multiple meta-analysis approaches are available and their relative strengths have not been directly compared using experimental data in the context of different gene expression scenarios and studies with different degrees of relationship. This study investigates the complementary advantages of meta-analysis approaches to integrate information across studies, and further mine the transcriptome for genes that are associated with complex processes such as behavioral maturation in honey bees. Behavioral maturation and division of labor in honey bees are related to changes in the expression of hundreds of genes in the brain. The information from various microarray studies comparing the expression of genes at different maturation stages in honey bee brains was integrated using complementary meta-analysis approaches.

**Results:**

Comparison of lists of genes with significant differential expression across studies failed to identify genes with consistent patterns of expression that were below the selected significance threshold, or identified genes with significant yet inconsistent patterns. The meta-analytical framework supported the identification of genes with consistent overall expression patterns and eliminated genes that exhibited contradictory expression patterns across studies. Sample-level meta-analysis of normalized gene-expression can detect more differentially expressed genes than the study-level meta-analysis of estimates for genes that were well described by similar model parameter estimates across studies and had small variation across studies. Furthermore, study-level meta-analysis was well suited for genes that exhibit consistent patterns across studies, genes that had substantial variation across studies, and genes that did not conform to the assumptions of the sample-level meta-analysis. Meta-analyses confirmed previously reported genes and helped identify genes (e.g. *Tomosyn*, *Chitinase 5, Adar, Innexin 2, Transferrin 1*, *Sick*, *Oatp26F*) and Gene Ontology categories (e.g. purine nucleotide binding) not previously associated with maturation in honey bees.

**Conclusion:**

This study demonstrated that a combination of meta-analytical approaches best addresses the highly dimensional nature of genome-wide microarray studies. As expected, the integration of gene expression information from microarray studies using meta-analysis enhanced the characterization of the transcriptome of complex biological processes.

## Background

One goal of microarray studies is to identify transcripts that are regulated similarly across a variety of contexts. The integration of gene expression information from multiple microarray studies can enhance the characterization of gene expression profiles that are consistently expressed across experiments. The across-study integration of information can support a more accurate identification of transcriptome biomarkers, functional categories, and pathways associated with the process of interest than results from individual studies.

Typical integration of information from multiple microarray studies relies on a simple comparison of lists of genes within study considered to be differentially expressed at a predetermined statistical threshold [1]. This approach is a useful first step to combine information across studies. However, simple overlap of lists of genes can result in potentially biased conclusions for two reasons. First, genes that may exhibit the same pattern across studies, but do not surpass the minimum threshold within one or multiple studies, may not be detected by this approach. Second, genes that may exhibit differential expression in more than one study may not reach differential expression when all the data across studies is considered, because the variation across studies is greater than the variation within study.

The usefulness of meta-analysis in clinical [2] and microarray studies [3,4] has been widely investigated. Rhodes et al. [5] implemented a meta-analysis of microarray studies by computing a summary statistic that consisted in summing the log-transformed significance *P-*values calculated for each study using one-sided random permutation *t*-tests. Some meta-analyses of microarray experiments aim at identifying biomarkers with particular expression patterns instead of exploring all possible profiles [6,7]. Other meta-analysis implementations compare the list of top ranking predictive genes using parametric and non-parametric rank aggregation approaches [8,9]. Although the use of significance and rank-order metrics removes concerns of incomparable expression levels across experiments, these approaches did not consider the profile of expression. Zhang and Fenstermacher [10] proposed the identification of promising reporter genes using a rank-sum test statistic, and the combination of expression levels across studies using a linear index that is trained and tested across data sets. A similar approach implemented by Schneider et al. [11] confirms the expression signature of selected genes found in one data set on other data sets. This two-stage approach may result in loss of information across stages, and accurate training and validation requires a large number of studies. Conlon et al. [12] proposed to use an indicator variable for differential expression that is a function of the total number of genes. However, the number of genes can vary between studies, and the indicator did not take into account the sign of the differential expression.

Model-based meta-analysis is a suitable framework to conduct an objective, integrative, and comparative study of multiple related microarray gene expression experiments, and help better understand the transcriptome and genomic basis of complex traits. In model-based meta-analysis, linear models are used to combine indicators of expression patterns from individual studies (e.g. fold changes or differences between mean groups, standardized estimates, or normalized values) and associated test-statistics or functions used to evaluate the expression pattern across studies. Traditional meta-analytical approaches that combine estimates (known as study-level meta-analysis) or observations (known as sample-level meta-analysis) across studies in one single step offer a comprehensive solution to the simultaneous consideration of multiple studies [3]. These approaches have a solid and extensive theoretical framework, jointly model expression patterns and associated measures of uncertainty, and can be used to detect differentially expressed genes across studies or to identify biomarkers associated with the conditions of interest. In addition to providing a list of genes differentially expressed, results from meta-analysis approaches are customarily depicted in funnel plots that facilitate the interpretation of results. However, a small number of applications of model-based meta-analysis to the expression of thousands of genes have been reported [13,3], and none have compared the performance of alternative approaches for different gene expression scenarios and across studies with different levels of relationship. Choi et al. [13] considered the meta-analysis of standardized mean differences, or differences between the means of the condition levels divided by the pooled standard deviation. These Student's *t*-statistic values were computed for each study and combined using fixed and random effects meta-analyses. The difference between these approaches is that random effects meta-analysis accounts for heterogeneity across studies. Random effects meta-analysis was more appropriate than fixed effects meta-analysis when combining studies from different research groups that may have substantial inter-study variation [13]. Studies using simulated data sets have compared the advantages of different approaches under different circumstances that can be extrapolated to gene expression data sets. Tudur-Smith et al. [14] conducted a meta-analysis of 5 simulated trials and concluded the absolute bias and spread of the estimate of the parameter increased as the degree of heterogeneity across studies increased. For a fixed underlying value of the parameter, the absolute bias in the estimate of the residual variance used in hypothesis testing did not exhibited a systematic pattern for increasing values of simulated heterogeneity. Wu et al. [15] explored the power and consistency to detect linkage and association of loci to a disease with meta-analysis of *P-*values (Fisher's meta-analysis), and pooled raw data analysis using simulated data. This study showed that, under homogeneous conditions, the results from meta-analysis and pooled data analysis were similar with meta-analysis having minimal loss of power.

The honey bee (*Apis mellifera*) is a well-established model organism to study the genomic architecture of physiological, neurological, and behavioral maturation [16]. Worker bee behavioral maturation results at the colony level in an age-related division of labor; young bees work in the hive for the first two to three weeks of adult life (performing tasks including brood care or "nursing") and older bees forage [17]. These behavioral changes are also associated with profound physiological, neuroanatomical, and neurochemical changes. Gene expression studies targeting specific honey bee genotypes (e.g. strains or subspecies), time points, and environmental conditions (e.g. host colony composition) have demonstrated that behavioral maturation in honey bees is associated with simultaneous changes in expression of thousands of genes in the brain [17-21]. Denison and Raymond-Delpech [22] presented an extensive review of changes in gene expression that accompany the transition to foraging.

The goals of this study are 1) to demonstrate the complementary advantages of the meta-analytical approaches to objectively integrate information across studies, and identification of genes with consistent expression profiles across studies are demonstrated, and 2) to fully mine information from eight microarray studies that have characterized differences in brain gene expression between one-day-old and forager honey bees. The performance of three complementary approaches (overlap of lists of genes, study- and sample-level meta-analyses) to detect consistent differential expression on a genome-wide level were compared. Functional analysis was performed to support the results from meta-analysis. From a biological perspective, the objective assessment of the degree of agreement between studies can help identify common regulatory genes and pathways that could be responsible for simultaneous (parallel or orthogonal) fluctuation in the expression of some genes and the inalterability in the expression of other genes.

## Results

A total of 7734 transcripts were analyzed and a summary of the number of transcripts with significant (unadjusted raw *P-*value < 1 × 10^-3^) differential expression obtained from the individual-study, study-level, and sample-level meta-analyses is presented in Table 1. The significance threshold corresponded to an approximate false discovery rate-adjusted *P-*value < 0.1 in the within study analyses. A breakdown of the results by positive (transcript over-expressed in forager compared to one-day-old honey bees) and negative (transcript over-expressed in one-day-old compared to forager honey bees) significant differential expression is provided in Additional file 1. The individual studies correspond to eight independent microarray datasets that do not share samples or microarrays, and include brain gene-expression measurements from one-day-old and forager honey bees. The eight studies were also divided into Group1 and Group 2, corresponding to the two separate publications [20,21] in which the studies were first presented. The four studies in Group 1 correspond to four distinct species; *A. mellifera *(AM), *A. cerana *(AC), *A. dorsata *(AD) and, *A. florea *(AF) honey bees raised in colonies of the same species [21]. The four studies in Group 2 correspond to *A. mellifera *(*A*. *m*.) honey bees from two subspecies raised in two host colonies: *A. mellifera mellifera *honey bees raised in an *A. mellifera mellifera *(MM) host colony, *A. mellifera mellifera *honey bees raised in an *A. mellifera ligustica *(ML) host colony, *A. mellifera ligustica *honey bees raised in an *A. mellifera ligustica *(LL) host colony, and *A. mellifera ligustica *honey bees raised in an *A. mellifera mellifera *(LM) host colony. Thus, in addition to individual- and meta-analysis, results from the two sets of studies (Group 1: studies AC, AD, AF, AM, and Group 2: MM, ML, LM, LL) are also described.

**Table 1:** Detection of differential expression by analysis

	**Individual Analyses**^1^								**Meta-Analyses**	
	**AC**	**AD**	**AF**	**AM**	**LL**	**LM**	**ML**	**MM**	**Study**	**Sample**

**AC**	152^2^	9^3^	55	60	15	21	21	26	6	123
**AD**	4.3%^4^	65	9	12	5	5	8	6	3	42
**AF**	38.5%	13.8%	143	53	19	24	12	18	9	99
**AM**	39.5%	18.5%	37.1%	310	20	35	37	38	6	168
**LL**	9.9%	7.7%	13.3%	6.5%	422	86	76	78	7	111
**LM**	13.8%	7.7%	16.8%	11.3%	22.8%	377	73	80	7	114
**ML**	13.8%	12.3%	8.4%	11.9%	18.0%	19.4%	540	57	6	126
**MM**	17.1%	9.2%	12.6%	12.3%	18.9%	21.2%	13.8%	413	8	117
**Study**	18.8%	9.4%	28.1%	18.8%	21.9%	21.9%	18.8%	25.0%	32	22
**Sample**	80.9%	64.6%	69.2%	54.2%	26.3%	30.2%	23.3%	28.1%	68.8%	853

^1^AC: *Apis cerana *bees raised on an *Apis cerana *colony; AD: *Apis dorsata *bees raised on an *Apis dorsata *colony; AF: *Apis florea *bees raised on an *Apis florea *colony; AM: *Apis mellifera *bees raised on an *Apis *mellifera colony, LL: *Apis mellifera ligustica *bees raised on an *Apis mellifera ligustica *colony; LM: *Apis mellifera ligustica *bees raised on an *Apis mellifera mellifera colony*; ML: *Apis mellifera mellifera *bees raised on an *Apis mellifera ligustica *colony; MM: *Apis mellifera mellifera *bees raised on an *Apis mellifera mellifera *colony.

^2^Number of transcripts with differential expression (P-value < 1 × 10^-3^) within individual analyses, study-level standardized (Study), and sample-level (Sample) meta-analysis (diagonals).

^3^Upper off-diagonals are the number of transcripts identified differentially expressed in all pairs of analyses relative to the maximum number of significant transcripts that can overlap in both analyses.

^4^Lower off-diagonals are the percentage of transcripts identified differentially expressed in all pairs of analyses relative to the maximum number of significant transcripts that can overlap in both analyses.

### Comparison of individual-study and meta analyses

The number of transcripts with significant differential expression between one-day-old and forager honey bees obtained from the individual study analyses ranged from 65 to 540 (Table 1). The AD study had the lowest number of differentially expressed transcripts and lowest overlap with any other study. Excluding results from AD, the number of differentially expressed genes present in at least two studies in Group 1 (AC, AF, AM) was on average 55, and slightly lower than the number of genes in at least two studies in Group 2 that ranged between 73 and 86. The percentage of cDNA transcripts differentially expressed and overlapping among the studies ranged from 4.3% to 39.5% (average of 16.1%) relative to the number of cDNAs identified in one of the studies. The highest and lowest overlap of differentially expressed transcripts was found in the comparison of studies AC against AM and AD, respectively (Table 1). Using the number of differentially expressed transcripts between one-day-old and forager honey bees as an indicator of the similarity between studies, AC, AF, and AM in Group 1 are more similar to each other with an overlap ranging from 53 to 60 transcripts, or approximately 38% of the significant genes. In Group 2, all studies have a similar overlap in number of significant transcripts, ranging from 57 to 86, or approximately 20% of the significant genes. The number of differentially expressed transcripts found in studies from two different groups ranged from 12 to 38, excluding AD that also had the lowest overlap with studies from Group 2. Across groups of studies, MM, ML, and LM are next in proximity to AM with 38, 37, and 35 genes in common, respectively. The highest overlap of differentially expressed genes among studies from different Groups was found between *A. mellifera *AM and MM studies.

The study-level and sample-level meta-analyses were able to overcome the weak consistency among studies, identifying 32 and 853 transcripts with significant differential expression, respectively (Table 1). The overlap of transcripts with differential expression between the study-level and subject-level meta-analyses was 68.8% of the minimum number of transcripts identified among both analyses. The overlap of transcripts with significant differential expression between the individual-study analyses and the study-level meta-analysis ranged from 18.8% to 28.1% of the transcripts identified within study, excluding study AD that had an overlap of 9.4%. The overlap of transcripts with significant differential expression between individual studies and the sample-level meta-analysis ranged from 23.3% to 80.9%.

**Figure 1:**
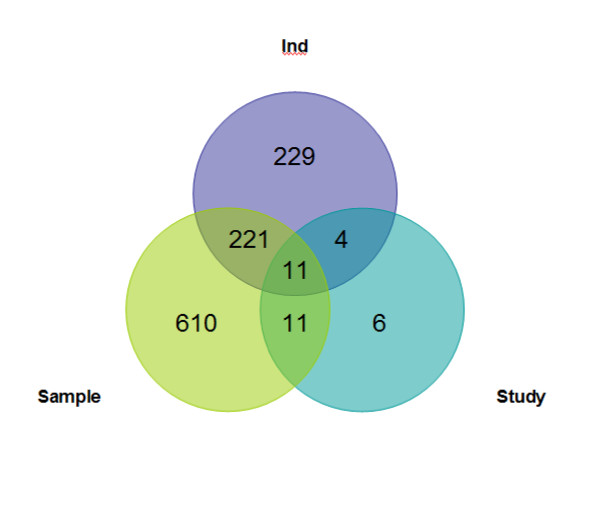
Venn diagram of the number of differentially expressed transcripts detected by at least two individual-study analyses (Ind), study-level (Study), and sample-level (Sample) meta-analyses.

The Venn diagram in Figure 1 depicts the overlap of transcripts identified as differentially expressed by the study-level and sample-level meta-analyses in at least two individual-study analyses. Of the 853 transcripts detected by the sample-level meta-analysis, 621 transcripts were not detected in at least two individual-study analyses, and 349 transcripts were not detected by any single individual-study analysis. Likewise, of the 32 transcripts detected by the study-level meta-analysis, 17 were not detected in at least two individual-study analyses. Of the 465 transcripts differentially expressed in the analysis of at least two studies, 236 were detected by meta-analysis, and 229 transcripts could not be confirmed by either the study- or sample-level meta-analyses. The differential expression of 11 transcripts was corroborated in all three cross-study approaches. The number of transcripts differentially expressed in two, three, and more studies was 320, 100, and 45 respectively (Additional file 2).

The number and percentage of transcripts detected by any individual-study analysis and sample-level meta-analysis is equal to or higher than the number of transcripts detected by the same individual-study analysis and study-level meta-analysis (Table 1). In addition, the relative percentage of genes that overlap between individual analyses and sample-level meta-analysis is substantially higher and more variable than the overlap with the study-level meta-analysis in all the Group 1 studies, relative to the Group 2 studies. The analysis of the AC study had the highest relative overlap of differentially expressed transcripts with sample-level meta-analysis among all individual-study analyses (80.9%), but had an intermediate overlap with the study-level meta-analysis results (18.8%) relative to other individual-study analyses. Conversely, the analysis of the ML study had the lowest overlap of transcripts differentially expressed with the sample-level meta-analysis among all individual-study analyses (23.3%), but had an intermediate overlap with the study-level meta-analysis (18.8%) relative to other individual-study analyses. The overlap between the transcripts detected to be differentially expressed by the individual analyses of Group 2 studies and study-level or sample-level meta-analyses were consistent across studies (Table 1).

### Consideration of gene expression profiles

Consideration of the sign (i.e. up- or down-regulation profile) of the transcripts detected by the individual and meta-analyses provided additional insights into the results (Additional file 1). There were no changes in the sign of transcripts detected as differentially expressed in two or more individual-study analyses within group, with the exception of 2 transcripts that had positive signs (over-expression in forager compared to one-day-old honey bees) in the analysis of the AM study, and had negative signs in the analysis of the AF study. In addition, a few transcripts exhibited changes in the expression pattern when studies from both groups were considered simultaneously. Amongst the Group 1 individual-study analyses, the AM study had the highest number of transcripts (14 transcripts) that had a different sign in at least one Group 2 individual-study analysis. The percentage of transcripts differentially expressed with the same sign and overlapping among the studies ranged from 1.8% to 43.2%, relative to the number of transcripts identified in one of the studies. The highest and lowest overlap of differentially expressed transcripts with the same sign was found in the comparison of the analysis of the AC study against the AM and AD studies, respectively (Additional file 1). The percentage of transcripts differentially expressed with different signs and overlapping among the studies ranged from 0% to 4.8%, relative to the number of transcripts identified in one of the studies. The analysis of the AD study resulted in the highest percentage of transcripts that had an opposite sign in any other individual-study analysis (4.8%), followed by the analysis of the AM study. The difference in ranking between absolute and relative counts is due to the fewer number of significant transcripts detected in the analysis of the AD study, compared to the AM study.

All the transcripts that exhibited differential expression in an individual-study analysis *and *study-level meta-analysis had a consistent pattern or sign (Additional file 1). Most transcripts that exhibited differential expression in an individual-study analysis *and *sample-level meta-analysis had a consistent pattern or sign. The transcripts that had a different sign in an individual-study analysis compared to the sample-level meta-analysis had the same sign as the meta-analysis in other individual-study analyses. Most analyses, individual-study and meta-study, had similar numbers of differentially expressed transcripts with positive (over-expressed in forager compared to one-day-old honey bees) and negative signs. In addition, no particular sign or pattern of differential expression dominated across analyses. The exception was study AM that had 97 and 213 positively and negatively differentially-expressed transcripts, respectively (Additional file 1). Although there were no substantial differences in the number of positive and negative estimates across all analyses considered, the overlap of counts by sign between individual analyses and sample-level meta-analysis did not necessarily reflect the trends observed in the individual analyses. This situation was observed in the LM, ML, and MM studies. For example, the total number of positive and negative results in ML was 263 and 277, respectively, yet the overlap with sample-level meta-analysis was 67 and 49, respectively (Additional file 1).

### Transcript meta-analysis scenarios

**Figure 2:**
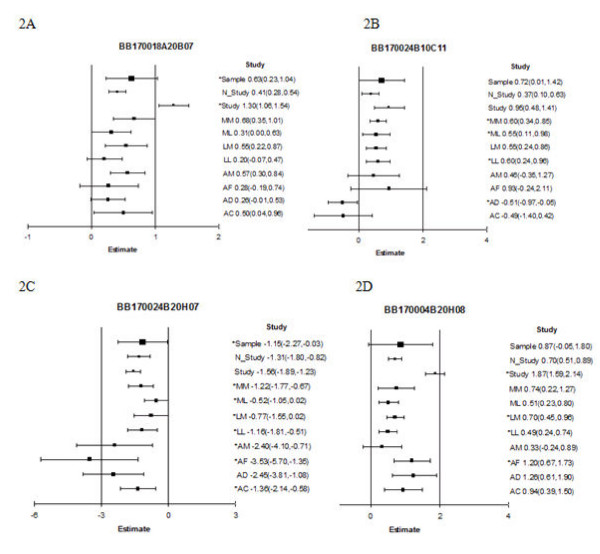
**Funnel plots of differential expression estimates and 95% confidence interval limits for *Apis mellifera *transcripts BB170018A20B07 (2A), BB170024B10C11 (2B), BB170024B20H07 (2C), and BB170004B20H08 (2D), by individual-study, study-level (Study), non-standardized study-level (N_Study), and sample-level (Sample) meta-analyses**. Estimates and 95% confidence intervals for each analysis are represented by a square and a horizontal line, respectively. Study denotes study-level meta-analysis of standardized estimates, N_Study denotes study-level meta-analysis of non-standardized estimates, Sample denotes sample-level meta-analysis. AC: *Apis cerana *bees raised on *Apis cerana *colonies; AD: *Apis dorsata *bees raised on *Apis dorsata *colonies; AF: *Apis florea *bees raised on *Apis florea *colonies; AM: *Apis mellifera *bees raised on an *Apis *mellifera colony, LL: *Apis mellifera ligustica *bees raised on an *Apis mellifera ligustica *colony; LM: *Apis mellifera ligustica *bees raised on an *Apis mellifera mellifera colony*; ML: *Apis mellifera mellifera *bees raised on an *Apis mellifera ligustica *colony; MM: *Apis mellifera mellifera *bees raised on an *Apis mellifera mellifera *colony The size of the square denoting the estimate corresponds to the number of observations in the study (AC, AD, AF n = 24; AM n = 22; LL, LM, ML, MM n = 12; study-level (Study) meta-analysis n = 8; sample-level (Sample) meta-analysis n = 142). Analyses detecting significant (P-value < 1 × 10^-3^) differential expression between forager and one-day-old honey bees are denoted by an asterisk.

To typify the strength of the different types of approaches to integrate information across studies, results from the individual-study, study-level and subject-level meta-analyses for scenarios of particular statistical and biological relevance were compared. Figure 2 presents the estimates (and 95% confidence intervals) of differential expression between forager and one-day-old honey bees corresponding to four transcript scenarios. Figure 2A depicts a transcript case where meta-analysis detected differential expression, meanwhile each individual-study analysis failed to detect differential expression. Both the sample-level and study-level meta-analyses detected differential expression for transcript BB170018A20B07, with significant differential expression across maturation stages. However, none of the eight individual-study analyses detected differential expression. This transcript represents the honey bee gene GB10350-PA that is similar to the fruit fly gene (FlyBase ID) FBgn0029997 (GO:0005515, protein binding molecular function), which encodes a protein reported to interact with the *Cyclin K *and muscle *LIM *proteins [23]. Figure 2B depicts a transcript (identifier BB170024B10C11) that exhibited differential expression in four individual-study analyses. Neither meta-analysis was able to detect this transcript because there was an inconsistent pattern of differential expression across the studies considered. This transcript represents the honey bee gene GB19001-PA that is similar to the fruit fly gene FBgn0032832 (*sick *or *sickie*), which encodes a protein reported to interact with a *pall (pallbearer) *protein [23]. Figure 2C depicts a transcript (identifier BB170024B20H01) that was differentially expressed in all except one individual-study analysis and the sample-level meta-analysis. However, the study-level meta-analysis was not able to uncover a significant difference. This transcript represents the honey bee gene GB13606-PA, predicted to code for a hypothetical protein. On the other hand, Figure 2D presents a transcript (identifier BB170004B20H08) that was consistently and differentially expressed in three individual-study analyses and in the study-level meta-analysis, but the sample-level meta-analyses was not able to uncover a significant difference. This transcript represents the honey bee gene GB15917-PA that is similar to the fruit fly gene FBgn0040351 (GO:0005509, calcium ion binding molecular binding), which encodes a protein reported to interact with the *Mephisto/Sickle *and the *KCNQ *potassium channel proteins [23].

### Genes and Gene Ontology classes

Individual genes and Gene Ontology [24] categories detected by the meta-analyses approaches were evaluated. Of the 863 unique transcripts with differential expression detected by either meta-analysis, 351 were new transcripts that would have not been detected in at least one individual-study analysis. Of the 351 transcripts, 347 transcripts were solely detected by the sample-level meta-analysis, two transcripts were solely detected by the study-level meta-analysis (identifiers BB160008A20A01, BB170022B20E11), and two additional transcripts (BB160014A10H01, BB170011B10F10) overlap between the study-level and sample-level meta-analyses. The latter four transcripts have not been assigned to honey bee genes or fruit fly gene orthologs.

The 347 transcripts detected by the sample-level meta-analysis mapped to 125 fruit fly genes with GO information (Additional file 3). The representation of molecular functions and biological processes among the 134 genes was analyzed using Fisher's exact test [25]. Genes for two molecular functions, cofactor binding (GO:0048037) and purine nucleotide binding (GO:0017076) had significant over-representation at P-value < 2.14 × 10^-3 ^and P-value < 7.04 × 10^-4^, respectively. Biological processes in which transcripts were significantly over-represented were macromolecule metabolic process (GO:0043170), cellular metabolic process (GO:0044237), and primary metabolic process (GO:0044238) with P-values < 2.86 × 10^-2^, 4.69 × 10^-2 ^and 6.57 × 10^-2^, respectively. Functional analysis of transcripts significant and differentially expressed from the sample-level meta-analysis that overlapped with any other individual-study or study-level meta-analyses identified enrichment of the following GO biological process categories: generation of precursor metabolites and energy (GO:0006091), cellular macromolecule metabolic process (GO:0044260), transport (GO:0006810), cell differentiation (GO:0030154), and system development (GO:0048731).

In terms of individual genes, among the 347 transcripts detected solely by the sample-level meta-analysis, two transcripts merit special attention because they are new to the list of genes associated with one-day-old and forager differences, and because of their known biological role. Transcript BB160024A10A12 similar to the fruit fly gene FBgn0026086 (*Adar*), was over-expressed in forager relative to one-day-old honey bees, and is involved in adult behavior (GO:0030534), adult locomotory behavior (GO:0008344), and response to heat (GO:0001666). Transcript BB170016B10A03, similar to fruit fly gene FBgn0027108 (*Innexin 2*), was also over-expressed in foragers and is involved in olfactory behavior (GO:0042048).

Forty-five genes were identified as differentially expressed in four or more studies, of which 39 appeared in one or both meta-analyses and 12 had GO information (Additional file 4). Genes over-expressed in foragers relative to one-day-old honey bees include genes corresponding to FlyBase IDs FBgn0038180, FBgn0051997 and FBgn0003036. Gene FBgn0038180 exhibits *Chitinase *activity and *Chitin *can be found in the exoskeleton of insects. FlyBase ID FBgn0003036, the *Para *gene, functions in male courtship behavior and veined wing generated song production. Conversely, genes under-expressed in foragers relative to one-day-old honey bees include FlyBase IDs FBgn0022355 (*Transferrin 1*, associated with defense response and ion transport activities), FBgn0035423 (associated with translation), FBgn0037146 (oxidoreductase activity), FBgn0038471 (methyltransferase activity), FBgn0038516 (oxidoreductase activity), FBgn0050035 (carbohydrate transmembrane transport activity) and FBgn0030412 (neurotransmitter secretion). Two genes had opposite differential expression patterns (signs) among studies. The *Sick *gene (Flybase ID FBgn0032832), that participates in the defense response to Gram-negative bacterium, was over-expressed in forager relative to one-day-old honey bees in studies LL, ML and MM, yet was under-expressed in forager relative to one-day-old honey bees in the study AD. This gene is represented as a funnel plot in Figure 2B. The *Oatp26F *gene (Flybase ID FBgn0051634) that participates in organic anion transportation, was over-expressed in forager relative to one-day-old honey bees in studies AC, AF and AM, yet under-expressed in forager relative to one-day-old honey bees in study MM.

## Discussion

### Comparison of individual-study and meta analyses

Figure 1 demonstrates the capability of meta-analysis to synergistically integrate consistent information across studies regardless of the significance level within study. Meta-analyses detected 627 differentially expressed transcripts that were not detected by overlap of lists of significant transcripts in at least two individual-study analyses. The failure of meta-analyses to detect 229 transcripts detected in at least two-individual analyses can be linked to contradictory or variability in the information provided by the individual studies. Although the relative overlap between the individual-study and study-level meta-analysis was always lower or equal than the overlap of the individual-study and sample-level meta-analysis, the variability of the overlap across studies further confirms that the advantages of the study- and sample-level meta-analysis depend not only on the transcript, but also on the studies being integrated (Table 1). The overlap in number of differentially expressed transcripts between the individual-study and meta-analyses approximately doubled relative to the values presented in Table 1 when the statistical significance threshold of the false discovery rate adjusted *P-*values was set to 0.2. The increment of the overlap was higher in the comparisons between individual-study and study-level meta-analysis (on average 2.3 fold) than in the comparisons between individual-study and sample-level meta-analysis (on average 1.9 fold). This slight difference may be due to the low sample size of the individual-study analyses and study-level meta-analyses that were more benefited by a less stringent significant threshold than the sample-level meta-analysis.

Transcripts with an inconsistent direction of differential expression or significance across the different meta-analyses require further study within and across studies. Adequate characterization of these possible scenarios can help design additional targeted experiments aimed at resolving the discrepancies among studies. The sample-level meta-analysis detected more differentially expressed transcripts than the study-level meta-analysis in cases with consistent expression profile across studies that exceeded potential variation across studies. The study-level approach was more appropriate to detect differential expression in transcripts with patterns that were consistent across studies, but less pronounced than the variation across studies. Because study is the experimental unit of the study-level meta-analysis, this approach may have insufficiently consistent and precise information to detect differential expression when a limited number of microarray experiments are available. A clear demonstration of the complementary advantage of the meta-analytical approaches across gene and study scenarios is provided in Figure 2. For example, Figure 2A illustrates the ability of the meta-analyses to combine consistent expression patterns of genes across studies and gain precision of estimates.

The imperfect overlap of results from the individual- and meta-study analyses corroborates reports that the association between the expression of numerous genes and behavioral maturation is highly sensitive to other genetic or environmental factors [22]. The complementary nature of the individual and meta-study approaches allows the identification of study-independent and study-dependent gene expression patterns. Evaluation of the overlap between individual-study analyses and sample-level meta-analysis provided insights into the multi-study scenarios that benefit the most from meta-analyses. For example, among Group 1 studies, the AM study had the highest overlap in number of significant transcripts with other studies, followed by AC and AF studies (Table 1). The same trend was observed in the overlap between these studies and the sample-level meta-analysis. On the other hand, the greater overlap in significant transcripts between Group 2 studies (plus the moderate overlap with Group 1 studies) relative to the overlap among Group 1 studies was not reflected in the overlap between Group 2 studies and the sample-level meta-analysis. An explanation for the seemingly contradictory behavior of sample-level meta-analysis and Group 1 versus Group 2 studies is the dimensionality of the overlap across more than two studies. Most of the overlaps between studies within Group 1 and other studies were generally observed only on pairs of studies, whereas most overlaps between studies within Group 2 and other studies were generally observed on more than two studies.

Another insight gained from the application of model-based meta-analysis approaches to the eight honey bee studies was the assessment of the variation in brain gene expression across studies, relative to the variation across honey bee genotypes. The reason for the higher overlap of differentially expressed genes among Group 2 studies, relative to the overlap among Group 1 studies, is that the samples pertain to *A. mellifera *subspecies while Group 1 studies corresponded to *A. mellifera *and other honey bee species. As expected, the transcripts identified in the analyses of the AM study in Group 1 exhibited the higher overlap with results from the Group 2 studies that used two *A. mellifera *subspecies (Table 1).

The transcripts detected in the analysis of the AC study had the highest relative overlap with sample-level meta-analysis and average overlap with the study-level meta-analysis results (Table 1). These results are consistent with the high overlap between the lists of transcripts detected by AC, AF and AM. The superior performance of the sample-level relative to the study-level meta-analysis for this study suggests that the analysis of the observations was able to pool the consistent information across studies that have low study-to-study variation. The combination of eight consistent estimates of differential expression in the study-level meta-analysis was not able to compensate for the number of estimates, and thus the overlap between the lists of transcripts from individual studies was lower. The inability of study-level meta-analysis to overcome partial consistency on a limited number of studies was also observed in the meta-analysis of mouse embryo studies [3].

### Consideration of gene expression profiles

Although the list of transcripts detected in the analysis of the AM study had high overlap with the lists of transcripts differentially expressed in the AC and AD studies, the AM list of transcripts had the lowest overlap with the sample-level meta-analysis list of genes. This result was related to the change in the sign or pattern of the differential expression of one-day-old relative to forager honey bees between the individual analysis and sample level meta-analysis (Additional file 1). The consideration of the overlap of significance *P-*values and sign of the estimates is critical to understanding this scenario. Although there was a high number of transcripts with significant differential expression in AM and AC or AD, the sign of some of these transcripts differed between these studies, and consequently, the meta-analysis did not detect these transcripts.

The analyses of AF and AD studies had the highest and lowest overlap of transcripts with study-level meta-analysis, respectively (Table 1). The former outcome suggests a situation where consistent estimates in AF and other studies, together with the low variation within study, overcomes the limited number of studies (eight estimates) analyzed, enhancing the capability of the study-level meta-analysis to detect differential expression. In this situation, the sample-level meta-analysis was unable to detect differential expression because the consistency of the results across studies was not able to compensate for the variation across studies. The outcome associated with the AD study is due to the low consistency of estimates between AD and other studies together with limited number of studies analyzed.

The sample-level meta-analysis detected more differentially expressed transcripts than the study-level meta-analysis in the presence of consistent patterns of expression in a few studies, and small variation between studies relative to the overall signal of differential expression. This is particularly evident on the ability of the sample-level meta-analysis to detect differentially expressed transcripts that overlap with individual studies, even though the signs may differ (Additional file 1). Transcripts with significant yet different expression patterns in one study and sample-level meta-analysis were detected in other studies with patterns consistent with the meta-analysis. There was no change in the sign of the profile of differential expression between individual analyses and study-level meta-analysis. Thus, the analysis of eight estimates by the study-level meta-analysis was not able to detect transcripts that may be significant in multiple studies but have different sign, for the levels of variation within study in the present work.

### Comparison to previous work

Results from the analyses of individual studies were consistent with results from the combined analysis of the AC, AD, AF, and AM studies presented by Sen Sarma et al. [21], and with results from the combined analysis of the MM, ML, LM, and LL data sets presented by Whitfield et al. [20] and Rodriguez-Zas et al. [17]. Sen Sarma et al. [21] reported a total of 1772 genes with differential expression (*P-*value < 1 × 10^-3^) between one day-old and forager honey bees across all four species (AC, AD, AF, AM), and of these, 218 genes were differentially expressed in two or more species. In the present analysis, 521 transcripts were differentially expressed in at least one species, and 113 transcripts were differentially expressed in two or more species in Group 1, also studied by Sen Sarma et al. [21] (Additional file 2). In the present study, a simple comparison of lists of differentially expressed genes indicated that AC and AM had the highest number of genes in common (Table 1). This result is consistent with the fact that, among all four species, AC and AM are most similar in ecological, physiological, and behavioral characteristics [21].

The difference on the total number of differentially expressed genes reported by Sen Sarma et al. [21] and in this study can be attributed to two reasons. First, the size of the data sets in the individual analyses presented in our study is approximately one-fourth of the total data set analyzed in Sen Sarma et al. [21]. The combined analysis of all four data sets is likely to offer more precise adjustments for technical sources of variation. Because a purpose of this study was to demonstrate the implementation of meta-analysis to integrate multiple studies, the four species were treated and analyzed as independent studies, thus potentially reducing the capability of each analysis to detect differentially expressed transcripts. Second, the model used to detect differential expression in Sen Sarma et al. [21] (including analysis of ratios using two ANOVAs) differed from the model considered in this study.

Similarly the lower number of genes with differential expression detected in any one individual analysis in the present study (1752 transcripts) compared to Whitfield et al. [20] (3745 genes) was attributed to different sample sizes and experimental models. Whitfield et al. [20] analyzed brain gene-expression measurements from 108 microarrays including one-day-old, forager, and honey bees at four additional intermediate stages of development, all arranged in a loop design. In the present study, only data from one-day-old and forager honey bees were compared, thus reducing the size of the data analyzed and information available to adjust for technical sources of variation. This difference in data sets was accompanied by a difference in the model used by Whitfield et al. [20].

The higher number of genes exhibiting differential expression between one-day-old and forager honey bees found in Group 2 studies relative to Group 1 studies is consistent with the higher number of differentially expressed genes reported by Whitfield et al. [20] relative to the number reported by Sen Sarma et al. [21] (Additional file 2). In the present work, the analyses of the LL and LM studies, that shared the subspecies of bee and differ on the colony, had the highest number of differentially expressed genes that overlapped (Table 1). This result is in agreement with Rodriguez-Zas et al. [17] that reported that the vast majority of the genes with differential expression across six time points in *A. mellifera ligustica *honey bees exhibited the same pattern across colonies.

The higher overlap of differentially expressed transcripts observed between pairs of Group 2 studies was expected, as all the samples corresponded to closely related honey bees subspecies (*A. mellifera mellifera *and *A. mellifera ligustica*). Also, the highest overlap of differentially expressed genes among studies from different Groups was found between AM and MM, followed by AM and ML. This result reflects that samples from the same bee species (*A. mellifera*) were used in both Groups, and confirms the stronger association of the bee species relative to the colony species on the gene expression patterns [20]. Results from the present studies also identified similarities between AM and LM that share the same colony subspecies (*A. mellifera*). This similarity may be due to the adjustment or adaptation of *A. mellifera ligustica *bees to the *A. mellifera mellifera *host colony. The reason for the limited overlap between genes differentially expressed across groups of studies, even within honey bee species, may be the use of different populations and environments. The four species of honey bees used by Sen Sarma et al. [21] were all collected from suburban areas of Bangalore, while the honey bees in Group 2 [20] were collected in France.

### Gene Ontology classes and individual genes

Gene Ontology analysis identified enrichment of generation of precursor metabolites and energy, cellular macromolecule metabolic process, transport, cell differentiation, system development, cofactor binding and purine nucleotide binding. These results were consistent with Sen Sarma et al. [21] that identified enrichment of numerous GO categories including protein binding, ion binding, and nucleic acid binding functions, response to biotic and abiotic factors, metabolism, pigmentation, and regulation of circadian rhythm, among others. Functional analysis of "hive to forager" genes found by Whitfield et al. [20], with hive encompassing 0-, 4-, 8-, 12-, and 17-day old bees, identified enrichment of genes associated with energy pathway physiological processes.

Among the transcripts only found differentially expressed in the sample-level meta-analysis, transcripts corresponding to the fruit fly genes *Adar *and *Innexin 2 *were over-expressed in forager relative to one-day-old honey bees. The *Adar *gene is associated with adult locomotory behavior and *Innexin 2 *is associated with olfactory behavior, both behaviors critical for honey bee foraging.

Consistent with Sen Sarma et al. [21] and Whitfield et al. [20], the expression of the honey bee orthologs to *the fruit fly *genes *Tctp *and *PebIII *was lower in forager compared to one-day-old honey bees, but the differential expression was only significant for *Tctp*. Whitfield et al. [20] reported a list of candidate genes for honey bee behavioral maturation that span all six maturation stages (0, 4, 8, 12, 17 day-old nurse and 17 day-old forager honey bees) considered. Out of this list, the percentages of transcripts differentially expressed (*P-*value < 1 × 10^-3 ^or borderline) in two or more individual-study analyses in the present work and in at least one species studied by Sen Sarma et al. [21] were 70% and between 58 and 75%, respectively. This result suggests that the results from the meta-analysis are supported by a previous independent study, and reiterates the superior ability of meta-analysis to detect differentially expressed transcripts compared to individual studies (i.e. 70% vs 58%). Among the genes detected in Whitfield et al. [20] and by meta-analysis in the present study are *Inos*, *Cah1*, *Hsc70cb*, *Mlc-c*, *Bm-40-spa*, *Zormin*, *Smd3*, *Tctp*, *Orc1*, *Ef2b*, *Sh3beta*, *PebIII*, *Rfabp*, *Fax*, and *Mmpp2*, and the FlyBase IDs were FBgn0050387 (receptor signaling protein), FBgn0037146 (glutamate 5-kinase), FBgn0037303 (cysteine protease inhibitor), FBgn0037140 (organic cation porter).

### Model extensions

The effect of behavior on gene expression may vary with other (secondary) factors or covariates. The interaction between the main (behavior) and secondary factors may originate at the study or sample level. Study-level factors influence all samples within a study in a similar fashion; meanwhile sample-level factors can have variable effects among honey bee samples. Study-level secondary variables can be included in the meta-analysis model. In this study, adjustment for secondary study-level factors, like study group, can be incorporated into the individual or traditional estimate-based meta-analysis because there are multiple studies per level of secondary factor. Group represents a study-level covariate, and thus, unbiased estimates can be obtained even in the presence of maturation stage heterogeneity across studies [2]. Colony is an example of a within-study source of variation that can also be included in the meta-analysis model. Group was not included in the present meta-analysis because the main goal was to investigate the benefits of meta-analysis expected in most situations, and because of the limited number of studies (four) per group. The availability of multiple studies per group that would allow an accurate adjustment for group effects is rare. In addition, the honey bee sample effect included in the individual-study and sample-level meta-analysis models also accounted for the previous sources of variation.

## Conclusion

The objective combination of information implemented in the complementary meta-analytical approaches was able to mine the signal of differential expression of data in different scenarios. Model-based meta-analysis approaches can rise above seemingly weak consistency among studies based on simple comparison of lists of genes. The sample-level meta-analysis detected more differentially expressed transcripts than the study-level meta-analysis among transcripts with consistent patterns of expression in a few studies, transcripts with expression well-described by similar model parameter estimates across studies, and transcripts with low variation between studies relative to the overall signal of differential expression. Study-level meta-analysis is the appropriate approach when only estimates of differences in expression among conditions of interest are available. Genes that do not conform to the assumptions of the sample-level meta-analysis, and have consistent expression patterns but substantial variation across studies, are benefited by the study-level meta-analysis.

Meta-analytical approaches uncovered genes associated with differences between one-day-old and forager honey bees across studies, regardless of the species or sub-species of honey bee sampled or in the colony. Among these, genes *Adar*, *Innexin 2*, *Chitin*, *Para, Transferrin 1*, *Sick*, *Oatp26F*, FBgn0022355, FBgn0035423, FBgn0037146, FBgn0038471, FBgn0038516, FBgn0050035, FBgn0030412, FBgn0032832, and FBgn0051634 are strong candidates for additional studies.

## Methods

### Data Sets

Eight microarray gene expression studies from two experiments were available for the meta-analysis. Four studies, hereby denoted Group 1 studies, are described in Sen Sarma et al. [21] and encompass the comparison of one-day-old and forager honey bees from four distinct species; *Apis mellifera *(AM), *A. cerana *(AC), *A. dorsata *(AD) and, *A. florea *(AF) raised in colonies of the same species. The remainder four gene expression studies, hereby denoted Group 2 studies, are described in Whitfield et al. [20] and Rodriguez-Zas et al. [17]. These studies encompassed comparisons of one-day-old and forager *Apis mellifera *(*A*. *m*.) honey bees from different subspecies. These comparisons included *A. mellifera mellifera *honey bees raised on an *A. mellifera mellifera *(MM) host colony, *A. mellifera mellifera *honey bees raised on an *A. mellifera ligustica *(ML) host colony, *A. mellifera ligustica *honey bees raised on an *A. mellifera ligustica *(LL) host colony, and *A. mellifera ligustica *honey bees raised on an *A. mellifera mellifera *(LM) host colony. The honey bee species *A. mellifera *and *A. cerana *are closer to each other than to *A. dorsata *and *A. florea *[26,27]. Meta-analysis of these studies can improve the detection of genes that are consistently over- (or under-) expressed in forager honey bees relative to one-day-old honey bees, regardless of the genetic make-up of the species or sub-species of the honey bee sampled or colony. Meta-analysis also supports the detection of genes that have unique expression patterns across species or within groups of species.

In all studies, forager honey bees were identified as honey bees that had pollen loads on hind legs. In Group 2 studies, forager honey bees could also have distended abdomens containing nectar or water load, and were 16-day to 17-day-old after adult emergence. The number of observations per study ranged from 12 (studies LL, LM, ML, and MM) to 24 (studies AC, AD, AF), and study AM had 22 observations. Due to variations in the size of the brain across honey bee species, brain samples were pooled (30 to 60 brain samples per pool) in the Group 1 studies. One-day-old and forager honey bees pertaining to the same colony (three colonies) were hybridized to the same microarray in a direct design with reverse labeling in all Group 1 studies. Direct comparison of one-day-old and forager honey bees was obtained using four microarrays (two microarrays per dye-labeling assignment) per colony for a total of 12 microarrays per study in Group 1. Individual brain samples were used in all Group 2 studies, each study including 20 microarrays in a loop design. Each 20-microarray loop corresponded to one combination of honey bee subspecies and colony (LL, LM, ML, and MM). One of the microarrays in all Group 2 studies included a direct comparison between one-day-old and forager honey bees, and an indirect comparison through 4 intermediate maturation stages [17]. Brain gene-expression data from all six stages in Group 2 studies was normalized together to improve the adjustment for technical variation. Only the normalized gene expression observations from one-day-old and forager honey bees were analyzed to make the models and analyses across all studies, regardless of group, comparable.

The expression of genes from individual brains was assessed using the double-spotted *A. mellifera *brain 9 K version 3.0 cDNA microarray [17]. A total of 5001 cDNA transcripts in the microarray were assigned to 3610 individual genes in the honey bee genome assembly version 2, and approximately 1970 genes have Gene Ontology information. Although an *A. mellifera *microarray was used to study different honey bee species and subspecies (AF, AD, AC, and *A. ligustica*), hybridization efficiency differences were minimal [20,21].

### Data processing and individual-study analysis

Data processing included the removal of spots that were flagged by the scanning software [28] or did not surpass a minimum threshold of 200, and log_2 _transformation of the background-subtracted foreground intensities. Log-transformed values were normalized using a global LOWESS transformation [29] to remove dye bias within microarray, and duplicate spots within gene were averaged. Microarray elements that did not have observations in all samples were removed from analysis to ensure the availability of the maximum possible information to estimate the parameter. A two-stage approach was used to adjust for technical sources of variation [30]. In the first stage, global dye and microarray effects were removed across all microarray elements or cDNAs transcripts, and in the second-stage individual-study or meta-analyses were implemented.

The individual-study analysis encompassed the second-stage model that described each transcript within study with the fixed effects of dye, maturation stage (one-day-old or forager), and the random effect of honey bee sample. Sample (honey bee) effects were assumed to be identically and independently distributed (iid), and from a Normal distribution with mean zero and a common honey bee variance *σ*^2^_*b*_. This common honey bee variance was due to the fact that the limited number of observations per sample precluded the precise estimation of separate honey bee variances. A microarray effect was not included in the model due to the design of Group 2 studies that included two honey bee samples not present on multiple microarrays. The number of transcripts studied within study ranged from 7734 to 7737. The number of gene expression measurements available per gene, study, and maturation stage ranged from 6 (Group 2 studies) to 12 (Group 1 studies AC, AF and AM).

Three approaches that combine the information on expression patterns in one-day-old versus forager honey bees across studies and within transcript were considered. The approaches were: standard overlap of genes with significant differential expression across studies (detected by the individual-study analyses), study-level meta-analysis of expression contrast estimates (obtained by the individual-study analyses), and sample-level meta-analysis of transcript expression across studies.

### Study-level meta-analysis

The study-level meta-analysis approach is pertinent when estimates of expression between conditions of interest (i.e. forager and one-day-old stages), and not the raw or normalized measurements of gene expression intensity, are available. The study-level meta-analysis (or Study) combines summary measurements across studies. The summary measurements are the estimates of the difference in brain gene expression between forager and one-day-old honey bees obtained from the individual-study analyses, standardized by the corresponding standard error. A study-level meta-analysis of non-standardized estimates (N_Study) was implemented to facilitate the comparison of study-level meta-analysis to individual-study and sample-level meta-analysis because the standardized estimates are unit-less, and therefore the results of the N_Study would have the same units as the results from the other analyses. An advantage of the standardized study-level meta-analysis is that the estimates have already been adjusted for technical sources of variation (i.e. dye, microarray) during the individual-study analysis stage.

A hierarchical mixed effects model was used to incorporate all sources of variation associated with the study-level standardized estimates combined in the study-level meta-analysis. For each transcript, the study-specific estimate is described with an overall difference in expression effect (*μ*), and the random effect of study (*s*_i_):

(1) $$\begin{array}{c} y_{i}\sim N\left(\mu +s_{i},\sigma^{2}\right)\\ s\sim N\left(0,\Sigma \right) \end{array}$$

where *y*_*i *_is the (standardized) estimate of difference in expression (forager versus one-day-old) obtained from the analysis of the *ith *study (*i *= 1 to 8), *ρ*^2 ^is the error variance, and ***s ***is the vector of study effects. Study was assumed to have a Normal distribution with mean zero and variance-covariance matrix ∑ of dimension k × k, where k is eight, the number of studies. The variance-covariance matrix had a diagonal structure with off-diagonals of zero, and diagonals representing the variance of the estimate of differential expression within study. The study-level meta-analysis model accounted for potential heterogeneity of variance among studies, and this is particularly important when combining information from potentially different subspecies. Meanwhile the standardized estimates of differential expression (*y*_*i*_) are adjusted for all other sources of variation fitted in the within-study model (i.e. dye and array effects), and standardized by the standard error of the estimate. In comparison, Choi et al. [13] considered the meta-analysis of differences between the means of the condition levels standardized by the pooled standard deviation.

### Sample-level meta-analysis

Like the individual-study analyses, the sample-level meta-analysis encompassed the second-stage model describing each transcript with the mixed effects model:

(2) $$\begin{array}{c} y_{ijklm}\sim N\left(\mu +d_{i}+m_{j}+s_{k}+b{\left(s\right)}_{kl},\sigma^{2}\right)\\ s\sim N\left(0,U\right)\\ b_{kl}\sim iidN\left(0,\sigma_{b}^{2}\right) \end{array}$$

where *y*_*ijklm *_is the normalized log-transformed brain gene expression corresponding to *mt*h observation, from the *lth *honey bee sample, at the *jth *maturation stage, pertaining to the *kth *study, labeled with the *ith *dye. Study effect was assumed to have a Normal distribution with mean zero and diagonal variance structure ***U ***of dimension k × k with a potentially different variance for each study. Sample effects were assumed to be *iid *and from a Normal distribution with mean zero and a common microarray variance *σ*^2^_*b*_, because of the limited number of observations per sample. Microarray was not included in the model so the sample-level meta-analysis model would be as similar as possible to the individual-study analysis model. A total of 142 observations were analyzed.

### General analysis considerations

Individual-study, study-level, and sample-level meta-analysis estimates were obtained using a restricted maximum-likelihood approach, and implemented using the SAS mixed procedure [31]. An experiment-wise type I error rate *α *= 1 × 10^-3 ^was used to identify transcripts differentially expressed across maturation stages. This significance threshold is equal to the threshold used by Sen Sarma et al. [21] and Whitfield et al. [20]. This threshold also proved to be a good compromise between the number of differentially expressed genes detected and the type I error rate while allowing to explore the overlap of a substantial number of genes across analyses. The analyses were compared in terms of overall number of transcripts identified as differentially expressed and the direction (sign) of the differential expression (over or under expressed in forager versus one-day-old honey bees).

The meta-analyses results of 7734 transcripts allowed the identification and characterization of different gene expression scenarios that benefited from different meta-analytical approaches. These scenarios were further explored using funnel plots that depict the estimate of differential expression between forager and one-day-old honey bees, and the associated confidence interval obtained in the individual-study, study-level, and sample-level meta-analysis. Four transcript cases were of particular statistical and biological relevance. First, transcripts that were not found differentially expressed in any individual study, yet were detected by the study-level or sample-level meta-analyses. Second, transcripts that had differential expression in opposite directions across studies and were not identified in the study-level or sample-level meta-analyses. Third, transcripts that were found differentially expressed in multiple individual studies and the sample-level meta-analysis, but were not detected by the study-level meta-analysis. Fourth, transcripts that were found differentially expressed in multiple individual studies and the study-level meta-analysis, but were not detected by the sample-level meta-analysis. Adequate characterization of these possible scenarios can help design additional targeted experiments to resolve the discrepancies among studies.

Results from the study-level and sample-level meta-analyses were further examined using functional enrichment analysis. Lists of differentially expressed genes were assigned to Gene Ontology (GO) biological processes and molecular functions classes based on fruit fly annotations. Representation of genes in GO classes was evaluated using Fisher's exact (two-tailed) test and False Discovery Rate multiple test adjustment [25].

## Authors' contributions

HAA performed the individual-study and meta-analyses, functional analysis of the results, contributed to the interpretation of results, and drafted the manuscript. BRS participated in the individual-study and meta-analyses and contributed to writing the manuscript. GER directed the microarray experiments that were meta-analyzed, helped interpret the results, and reviewed the manuscript. SRZ obtained funding for the study, participated in its conception, coordination, interpretation of results, and helped write the manuscript. All authors have read and approved the final version of this manuscript.

## Supplementary Material

Additional file 1 **Detection of differential expression by direction and analysis**. Number of transcripts with positive (over-expression in forager compared to one-day-old honey bees) and negative (under-expression in forager compared to one-day-old honey bees) differential expression (P-value < 1 × 10^-3^) within individual analysis, study-level standardized (Study) and sample-level (Sample) meta-analysis (diagonals); number (upper off-diagonals), and percentage (lower off-diagonals) of transcripts identified differentially expressed in all pairs of analyses relative to the maximum number of significant transcripts that can overlap in both analyses. Click here for file

Additional file 2 **Table identifying the number of transcripts non-significant or significant within group, and total**. Number of transcripts with non-significant and significant (Sig., P-value < 1 × 10^-3^) differential expression between forager and one-day-old honey bees within studies in Group 1 (AC^1^, AD, AF, AM), Group 2 (LL, LM, ML, MM), and both groups combined. Click here for file

Additional file 3 **Gene Ontology information for transcripts identified in the sample-level meta-analysis**. Gene Ontology (molecular function or mol. function, biological process or bio. process, and cellular component or cell. component) and fruit fly information for 125 *Apis mellifera *transcripts with significant differential expression in only the sample-level meta-analysis. Click here for file

Additional file 4 **Gene Ontology information for transcripts identified in four or more studies**. Gene Ontology (molecular function or mol. function, biological process or bio. process, and cellular component or cell. component) information for 12 transcripts with significant differential expression in four or more studies. Click here for file
