# Supplementary material for: Meta-analysis of genome-wide expression patterns associated with behavioral maturation in honey bees
Source: BMC Genomics. 2008 Oct 24;9:503. doi: 10.1186/1471-2164-9-503 (PMC2582039; doi:10.1186/1471-2164-9-503)
Supplement: Additional file 1 — Detection of differential expression by direction and analysis. Number of transcripts with positive (over-expression in forager compared to one-day-old honey bees) and negative (under-expression in forager compared to one-day-old honey bees) differential expression (P-value < 1 × 10^-3^) within individual analysis, study-level standardized (Study) and sample-level (Sample) meta-analysis (diagonals); number (upper off-diagonals), and percentage (lower off-diagonals) of transcripts identified differentially expressed in all pairs of analyses relative to the maximum number of significant transcripts that can overlap in both analyses. [file 1471-2164-9-503-S1.doc]

## Additional file 1

**Detection of differential expression by direction and analysis.**

|  |  | **Individual Analyses**^1^ | | | | | | | | | | | | | | | | **Meta-Analyses** | | | |  |
| --- | --- | --- | --- | --- | --- | --- | --- | --- | --- | --- | --- | --- | --- | --- | --- | --- | --- | --- | --- | --- | --- | --- |
|  |  | **AC** | | **AD** | | **AF** | | **AM** | | **LL** | | **LM** | | **ML** | | **MM** | | **Study** | | **Sample** | |  |
|  |  | **+** | **-** | **+** | **-** | **+** | **-** | **+** | **-** | **+** | **-** | **+** | **-** | **+** | **-** | **+** | **-** | **+** | **-** | **+** | **-** |  |
| **AC** | **+** | 59^2^ | - | 6^3^ | 0 | 22 | 0 | 20 | 0 | 3 | 0 | 7 | 0 | 7 | 0 | 7 | 1 | 2 | 0 | 39 | 10 | |
|  | **-** | - | 93 | 0 | 3 | 0 | 33 | 0 | 40 | 0 | 12 | 0 | 14 | 1 | 13 | 0 | 18 | 0 | 4 | 7 | 67 | |
| **AD** | **+** | 13.6%^4^ | 0 | 44 | - | 6 | 0 | 7 | 0 | 2 | 0 | 3 | 0 | 2 | 2 | 5 | 0 | 2 | 0 | 22 | 4 | |
|  | **-** | 0.0% | 14.3% | - | 21 | 0 | 3 | 0 | 5 | 1 | 2 | 0 | 2 | 1 | 3 | 1 | 0 | 0 | 1 | 4 | 12 | |
| **AF** | **+** | 40.0% | 0.0% | 13.6% | 0.0% | 55 | - | 13 | 0 | 6 | 0 | 8 | 0 | 3 | 0 | 1 | 1 | 3 | 0 | 30 | 7 | |
|  | **-** | 0.0% | 37.5% | 0.0% | 14.3% | - | 88 | 2 | 38 | 0 | 13 | 1 | 15 | 0 | 9 | 2 | 14 | 0 | 6 | 7 | 55 | |
| **AM** | **+** | 33.9% | 0.0% | 15.9% | 0.0% | 23.6% | 2.3% | 97 | - | 4 | 0 | 9 | 1 | 11 | 3 | 6 | 2 | 4 | 0 | 37 | 11 | |
|  | **-** | 0.0% | 43.0% | 0.0% | 23.8% | 0.0% | 43.2% | - | 213 | 0 | 16 | 1 | 24 | 3 | 20 | 4 | 26 | 0 | 2 | 26 | 94 | |
| **LL** | **+** | 5.1% | 0.0% | 4.5% | 4.8% | 10.9% | 0.0% | 4.1% | 0.0% | 238 | - | 46 | 0 | 48 | 0 | 45 | 0 | 3 | 0 | 57 | 1 | |
|  | **-** | 0.0% | 12.9% | 0.0% | 9.5% | 0.0% | 14.8% | 0.0% | 8.7% | - | 184 | 0 | 40 | 0 | 28 | 0 | 33 | 0 | 4 | 4 | 49 | |
| **LM** | **+** | 11.9% | 0.0% | 6.8% | 0.0% | 14.5% | 1.1% | 9.3% | 0.5% | 23.0% | 0.0% | 200 | - | 41 | 0 | 32 | 0 | 3 | 0 | 53 | 1 | |
|  | **-** | 0.0% | 15.1% | 0.0% | 9.5% | 0.0% | 17.0% | 1.0% | 13.6% | 0.0% | 22.6% | - | 177 | 0 | 32 | 0 | 48 | 0 | 4 | 0 | 60 | |
| **ML** | **+** | 11.9% | 1.1% | 4.5% | 4.8% | 5.5% | 0.0% | 11.3% | 1.4% | 20.2% | 0.0% | 20.5% | 0.0% | 263 | - | 34 | 0 | 3 | 0 | 67 | 4 | |
|  | **-** | 0.0% | 14.0% | 4.5% | 14.3% | 0.0% | 10.2% | 3.1% | 9.4% | 0.0% | 15.2% | 0.0% | 18.1% | - | 277 | 0 | 23 | 0 | 3 | 6 | 49 | |
| **MM** | **+** | 11.9% | 0.0% | 11.4% | 4.8% | 1.8% | 2.3% | 6.2% | 1.9% | 21.5% | 0.0% | 16.0% | 0.0% | 16.3% | 0.0% | 209 | - | 4 | 0 | 49 | 0 | |
|  | **-** | 1.7% | 19.4% | 0.0% | 0.0% | 1.8% | 15.9% | 2.1% | 12.7% | 0.0% | 17.9% | 0.0% | 27.1% | 0.0% | 11.3% | - | 204 | 0 | 4 | 0 | 68 | |
| **Study** | **+** | 12.5% | 0.0% | 12.5% | 0.0% | 18.8% | 0.0% | 25.0% | 0.0% | 18.8% | 0.0% | 18.8% | 0.0% | 18.8% | 0.0% | 25.0% | 0.0% | 16 | - | 11 | 0 | |
|  | **-** | 0.0% | 25.0% | 0.0% | 6.3% | 0.0% | 37.5% | 0.0% | 12.5% | 0.0% | 25.0% | 0.0% | 25.0% | 0.0% | 18.8% | 0.0% | 25.0% | - | 16 | 0 | 11 | |
| **Sample** | **+** | 66.1% | 7.5% | 50.0% | 19.0% | 54.5% | 8.0% | 38.1% | 12.2% | 23.9% | 2.2% | 26.5% | 0.0% | 25.5% | 2.2% | 23.0% | 0.0% | 68.8% | 0.0% | 437 | - | |
|  | **-** | 16.9% | 72.0% | 9.1% | 57.1% | 12.7% | 62.5% | 11.3% | 44.1% | 0.4% | 26.6% | 0.5% | 33.9% | 1.5% | 17.7% | 0.0% | 33.3% | 0.0% | 68.8% | - | 416 | |

^1^AC: *Apis* *cerana* bees raised on *an Apis cerana* colony; AD: *Apis* *dorsata* bees raised on an *Apis dorsata* colony; AF: *Apis* *florea* bees raised on an *Apis florea* colony; AM: *Apis* *mellifera* bees raised on an *Apis* mellifera colony, LL: *Apis* *mellifera* *ligustica* bees raised on an *Apis* *mellifera* *ligustica* colony; LM: *Apis* *mellifera* *ligustica* bees raised on an *Apis* *mellifera mellifera colony*; ML: *Apis* *mellifera* *mellifera* bees raised on a *Apis* *mellifera* *ligustica* colony; MM: *Apis* *mellifera* *mellifera* bees raised on an *Apis* *mellifera* *mellifera* colony.

^2^Number of transcripts with differential expression (P-value < 1x10^-3^) within individual analyses, study-level standardized (Study), and sample-level (Sample) meta-analysis (diagonals).

^3^Upper off-diagonals are the number of transcripts identified differentially expressed in all pairs of analyses relative to the maximum number of significant transcripts that can overlap in both analyses.

^4^Lower off-diagonals are the percentage of transcripts identified differentially expressed in all pairs of analyses relative to the maximum number of significant transcripts that can overlap in both analyses.
