# Supplementary material for: Meta-analysis of genome-wide expression patterns associated with behavioral maturation in honey bees
Source: BMC Genomics. 2008 Oct 24;9:503. doi: 10.1186/1471-2164-9-503 (PMC2582039; doi:10.1186/1471-2164-9-503)
Supplement: Additional file 2 — Table identifying the number of transcripts non-significant or significant within group, and total. Number of transcripts with non-significant and significant (Sig., P-value < 1 × 10^-3^) differential expression between forager and one-day-old honey bees within studies in Group 1 (AC1, AD, AF, AM), Group 2 (LL, LM, ML, MM), and both groups combined. [file 1471-2164-9-503-S2.doc]

## Additional file 2

**Number of transcripts with non-significant and significant (Sig., P-value < 1x10^-3^) differential expression between forager and one-day-old honey bees within studies in Group 1 (AC^1^, AD, AF, AM), Group 2 (LL, LM, ML, MM), and both groups combined.**

|  | **Group 1** | **Group 2** | **Both Groups** |
| --- | --- | --- | --- |
| **Non-significant** | 7213 | 3652 | 5978 |
| **Sig. in 1 study** | 408 | 1092 | 1294 |
| **Sig. in 2 studies** | 69 | 228 | 320 |
| **Sig. in 3 studies** | 39 | 56 | 100 |
| **Sig. in 4 studies** | 5 | 9 | 29 |
| **Sig. in 5 studies** | N/A | N/A | 12 |
| **Sig. in 6 studies** | N/A | N/A | 3 |
| **Sig. in 7 studies** | N/A | N/A | 1 |
| **Total** | 7734 | 7737 | 7737 |

^1^AC: *Apis* *cerana* bees raised on an *Apis cerana* colony; AD: *Apis* *dorsata* bees raised on an *Apis dorsata* colony; AF: *Apis* *florea* bees raised on an *Apis florea* colony; AM: *Apis* *mellifera* bees raised on an *Apis mellifera* colony, LL: *Apis* *mellifera* *ligustica* bees raised on an *Apis* *mellifera* *ligustica* colony; LM: *Apis* *mellifera* *ligustica* bees raised on an *Apis* *mellifera mellifera colony*; ML: *Apis* *mellifera* *mellifera* bees raised on an *Apis* *mellifera* *ligustica* colony; MM: *Apis* *mellifera* *mellifera* bees raised on an *Apis* *mellifera* *mellifera* colony.
