# Supplementary material for: Meta-analysis of genome-wide expression patterns associated with behavioral maturation in honey bees
Source: BMC Genomics. 2008 Oct 24;9:503. doi: 10.1186/1471-2164-9-503 (PMC2582039; doi:10.1186/1471-2164-9-503)
Supplement: Additional file 3 — Gene Ontology information for transcripts identified in the sample-level meta-analysis. Gene Ontology (molecular function or mol. function, biological process or bio. process, and cellular component or cell. component) and fruit fly information for 125 Apis mellifera transcripts with significant differential expression in only the sample-level meta-analysis. [file 1471-2164-9-503-S3.doc]

## Additional file 3

**Gene Ontology (molecular function or mol. function, biological process or bio. process, and cellular component or cell. component) and fruit fly information for 125 *Apis* *mellifera*** transcripts with significant differential expression in only the sample-level meta-analysis.

| ***Transcript*** | ***Computed Gene (CG) Symbol*** | ***FlyBase ID*** | ***Gene Name*** | ***GO*** | ***GO Category*** | ***GO Description*** |
| --- | --- | --- | --- | --- | --- | --- |
| BB160003A20B08 | CG10882 | FBgn0031408 |  | GO:0005811 | Cell. Component | lipid particle |
|  |  |  |  | GO:0004252 | Mol. Function | serine-type endopeptidase activity |
| BB160003B20H02 | CG33276 | FBgn0053276 |  | GO:0006508 | Bio. Process | proteolysis |
|  |  |  |  | GO:0005576 | Cell. Component | extracellular region |
|  |  |  |  | GO:0004295 | Mol. Function | trypsin activity |
| BB160004B10F12 | CG5014 | FBgn0029687 | Vap-33-1 | GO:0007528 | Bio. Process | neuromuscular junction |
|  |  |  |  | GO:0007269 | Bio. Process | neurotransmitter secretion |
|  |  |  |  | GO:0016082 | Bio. Process | synaptic vesicle priming |
|  |  |  |  | GO:0005811 | Cell. Component | lipid particle |
|  |  |  |  | GO:0008021 | Cell. Component | synaptic vesicle |
| BB160006A10D08 | CG5119 | FBgn0003031 | pAbp polyA-binding protein | GO:0045727 | Bio. Process | positive regulation of translation |
|  |  |  |  | GO:0007268 | Bio. Process | synaptic transmission |
|  |  |  |  | GO:0005737 | Cell. Component | cytoplasm |
|  |  |  |  | GO:0005811 | Cell. Component | cytoplasm |
|  |  |  |  | GO:0003729 | Mol. Function | mRNA binding |
|  |  |  |  | GO:0008143 | Mol. Function | poly(A) binding |
|  |  |  |  | GO:0005515 | Mol. Function | protein binding |
| BB160006A20A01 | CG9894 | FBgn0031453 |  | GO:0005634 | Cell. Component | nucleus |
| BB160006A20F11 | CG6560 | FBgn0038916 |  | GO:0003924 | Mol. Function | GTPase activity |
| BB160006B20E08 | CG5166 | FBgn0041188 | Atx2 Ataxin-2 | GO:0007015 | Bio. Process | actin filament organization |
|  |  |  |  | GO:0022416 | Bio. Process | bristle development |
|  |  |  |  | GO:0048749 | Bio. Process | compound eye development |
|  |  |  |  | GO:0009994 | Bio. Process | oocyte differentiation |
|  |  |  |  | GO:0006911 | Bio. Process | phagocytosis, engulfment |
|  |  |  |  | GO:0030833 | Bio. Process | regulation of actin filament polymerization |
|  |  |  |  | GO:0005737 | Cell. Component | cytoplasm |
| BB160006B20G06 | CG18783 | FBgn0028420 | Kr-h1, Kruppel homolog 1 | GO:0007552 | Bio. Process | metamorphosis |
|  |  |  |  | GO:0045449 | Bio. Process | regulation of transcription |
|  |  |  |  | GO:0035075 | Bio. Process | response to ecdysone |
|  |  |  |  | GO:0005634 | Cell. Component | nucleus |
|  |  |  |  | GO:0003700 | Mol. Function | transcription factor activity |
| BB160007B10H07 | CG4119 | FBgn0028474 |  | GO:0000381 | Bio. Process | regulation of alternative nuclear mRNA splicing, via spliceosome |
|  |  |  |  | GO:0005634 | Cell. Component | nucleus |
|  |  |  |  | GO:0003729 | Mol. Function | mRNA binding |
| BB160007B20G10 | CG5972-PA | FBgn0031781 | Arc-p20 | GO:0030031 | Bio. Process | cell projection biogenesis |
|  |  |  |  | GO:0030866 | Bio. Process | cortical actin cytoskeleton organization |
|  |  |  |  | GO:0006911 | Bio. Process | phagocytosis, engulfment |
|  |  |  |  | GO:0008360 | Bio. Process | regulation of cell shape |
|  |  |  |  | GO:0003779 | Mol. Function | actin binding |
| BB160008B10A08 | CG6170 | FBgn0026428 | HDAC6 | GO:0016575 | Bio. Process | histone deacetylation |
|  |  |  |  | GO:0004407 | Mol. Function | histone deacetylase activity |
| BB160010A20A10 | CG3376 | FBgn0034997 |  | GO:0004767 | Mol. Function | sphingomyelin phosphodiesterase activity |
| BB160010A20D02 | CG11071 | FBgn0030532 |  | GO:0003700 | Mol. Function | transcription factor activity |
| BB160010A20D02 | CG32611 | FBgn0085375 | mamo maternal gene required for meiosis | GO:0007143 | Bio. Process | female meiosis |
|  |  |  |  | GO:0007276 | Bio. Process | gamete generation |
|  |  |  |  | GO:0035041 | Bio. Process | sperm chromatin decondensation |
|  |  |  |  | GO:0005737 | Cell. Component | cytoplasm |
|  |  |  |  | GO:0005634 | Cell. Component | nucleus |
|  |  |  |  | GO:0003676 | Mol. Function | nucleic acid binding |
|  |  |  |  | GO:0003700 | Mol. Function | transcription factor activity |
| BB160011A10F02 | CG7610 | FBgn0020235 | ATPsyn-gamma ATP syntase-gamma chain | GO:0006911 | Bio. Process | phagocytosis, engulfment |
|  |  |  |  | GO:0015992 | Bio. Process | proton transport |
|  |  |  |  | GO:0005811 | Cell. Component | lipid particle |
|  |  |  |  | GO:0000275 | Cell. Component | mitochondrial proton-transporting ATP synthase complex, catalytic core F(1) |
|  |  |  |  | GO:0008553 | Mol. Function | hydrogen-exporting ATPase activity, phosphorylative mechanism |
| BB160011B10B09 | CG10546 | FBgn0035636 | Cralbp Cellular retinaldehyde binding protein | GO:0016918 | Mol. Function | retinal binding |
| BB160012B10C02 | CG4169 | FBgn0036642 |  | GO:0006122 | Bio. Process | mitochondrial electron transport, ubiquinol to cytochrome c |
|  |  |  |  | GO:0005811 | Cell. Component | lipid particle |
|  |  |  |  | GO:0005750 | Cell. Component | mitochondrial respiratory chain complex III |
|  |  |  |  | GO:0008121 | Mol. Function | ubiquinol-cytochrome-c reductase activity |
| BB160012B20B11 | CG18069 | FBgn0004624 | CaMKII Calcium/calmodulin-dependent protein kinase II | GO:0007619 | Bio. Process | courtship behavior |
|  |  |  |  | GO:0007611 | Bio. Process | learning and/or memory |
|  |  |  |  | GO:0007616 | Bio. Process | long-term memory |
|  |  |  |  | GO:0008049 | Bio. Process | male courtship behavior |
|  |  |  |  | GO:0007528 | Bio. Process | meuromuscular junction |
|  |  |  |  | GO:0006468 | Bio. Process | protein amino acid phosphorylation |
|  |  |  |  | GO:0051489 | Bio. Process | regulation of filopodium formation |
|  |  |  |  | GO:0007268 | Bio. Process | synaptic transmission |
|  |  |  |  | GO:0030424 | Cell. Component | axon |
|  |  |  |  | GO:0005954 | Cell. Component | calcium- and calmodulin- dependent protein kinase complex |
|  |  |  |  | GO:0030425 | Cell. Component | dendrite |
|  |  |  |  | GO:0045211 | Cell. Component | postsynaptic membrane |
|  |  |  |  | GO:0048786 | Cell. Component | presynaptic active zone |
|  |  |  |  | GO:0005516 | Mol. Function | calmodulin binding |
|  |  |  |  | GO:0004683 | Mol. Function | calmodulin-dependent protein kinase activity |
|  |  |  |  | GO:0004674 | Mol. Function | protein serine/threonine kinase activity |
| BB160012B20C06 | CG7828 | FBgn0036127 |  | GO:0004839 | Mol. Function | ubiquitin activating enzyme activity |
| BB160012B20E05 | CG10545 | FBgn0001105 | Gbeta13F G protein beta-subunit 13F | GO:0007015 | Bio. Process | actin filament organization |
|  |  |  |  | GO:0045176 | Bio. Process | apical protein localization |
|  |  |  |  | GO:0055059 | Bio. Process | asymmetric neuroblast division |
|  |  |  |  | GO:0007816 | Bio. Process | G-protein coupled receptor protein signaling pathway |
|  |  |  |  | GO:0005834 | Cell. Component | heterotrimeric G-protein complex |
|  |  |  |  | GO:0003924 | Mol. Function | GTPase activity |
| BB160013A10A12 | CG10743 | FBgn0036376 |  | GO:0008268 | Mol. Function | receptor signaling protein tyrosine kinase signaling protein activity |
| BB160013A10E06 | CG4088 | FBgn0005654 | lat latheo | GO:0006260 | Bio. Process | DNA replication |
|  |  |  |  | GO:0006270 | Bio. Process | DNA replication initiation |
|  |  |  |  | GO:0030536 | Bio. Process | larval feeding behavior |
|  |  |  |  | GO:0007612 | Bio. Process | learning |
|  |  |  |  | GO:0007611 | Bio. Process | learning and/or memory |
|  |  |  |  | GO:0008355 | Bio. Process | olfactory learning |
|  |  |  |  | GO:0005664 | Cell. Component | nuclear origin of replication recognition complex |
|  |  |  |  | GO:0003677 | Mol. Function | DNA binding |
| BB160013A20B05 | CG4356 | FBgn0000037 | mAcR-60C muscarinic Acetylcholine Receptor 60 C | GO:0007213 | Bio. Process | acetylcholine receptor signaling, muscarinic pathway |
|  |  |  |  | GO:0016021 | Cell. Component | integral to membrane |
|  |  |  |  | GO:0005887 | Cell. Component | integral to plasma membrane |
|  |  |  |  | GO:0005886 | Cell. Component | plasma membrane |
|  |  |  |  | GO:0008227 | Mol. Function | amine receptor activity |
|  |  |  |  | GO:0004981 | Mol. Function | muscarinic acetylcholine receptor activity |
| BB160013B10A02 | CG7762 | FBgn0028695 | Rpn1 | GO:0008283 | Bio. Process | cell proliferation |
|  |  |  |  | GO:0000022 | Bio. Process | mitotic spindle elongation |
|  |  |  |  | GO:0007052 | Bio. Process | mitotic spindle organization and biogenesis |
|  |  |  |  | GO:0006508 | Bio. Process | proteolysis |
|  |  |  |  | GO:0005838 | Cell. Component | proteasome regulatory particle |
|  |  |  |  | GO:0008540 | Cell. Component | proteasome regulatory particle, base subcomplex |
|  |  |  |  | GO:0004175 | Mol. Function | endopeptidase activity |
| BB160014A10F04 | CG5427 | FBgn0032433 | Oatp33Ea Organic anion transporting polypeptide 33Ea | GO:0015711 | Bio. Process | organic anion transport |
|  |  |  |  | GO:0008514 | Mol. Function | organic anion transmembrane transporter activity |
|  |  |  |  | GO:0005215 | Mol. Function | transporter activity |
| BB160014A10G06 | CG14437 | FBgn0029502 | COQ7 | GO:0006743 | Bio. Process | ubiquinone metabolic process |
|  |  |  |  | GO:0005743 | Cell. Component | mitochondrial inner membrane |
| BB160014A20H02 | CG14813 | FBgn0028969 | deltaCOP delta-coatomer protein | GO:0006911 | Bio. Process | phagocytosis, engulfment |
|  |  |  |  | GO:0006890 | Bio. Process | retrograde vesicle-mediated transport, Golgi to ER |
|  |  |  |  | GO:0030126 | Cell. Component | COPI vesicle coat |
| BB160015A20E08 | CG1417 | FBgn0003423 | slgA sluggish A | GO:0006537 | Bio. Process | glutamate biosynthetic process |
|  |  |  |  | GO:0007626 | Bio. Process | locomotory behavior |
|  |  |  |  | GO:0042331 | Bio. Process | phototaxis |
|  |  |  |  | GO:0006562 | Bio. Process | proline catabolic process |
|  |  |  |  | GO:0005759 | Cell. Component | mitochondrial matrix |
|  |  |  |  | GO:00046757 | Mol. Function | proline dehydrogenase activity |
| BB160016A10H10 | CG2152 | FBgn0015276 | Pcmt Protein-L-isoaspartate (D-aspartate) O-methyltransferase | GO:0006464 | Bio. Process | protein modification process |
|  |  |  |  | GO:0030091 | Bio. Process | protein repair |
|  |  |  |  | GO:0005737 | Cell. Component | cytoplasm |
|  |  |  |  | GO:0004719 | Mol. Function | protein-L-isoaspartate (D-aspartate) O-methyltransferase activity |
| BB160016B10A11 | CG6281 | FBgn0025879 | Timp Tissue inhibitor of metalloproteases | GO:0030425 | Cell. Component | dendrite |
|  |  |  |  | GO:0005576 | Cell. Component | extracellular region |
|  |  |  |  | GO:0008191 | Mol. Function | metalloendopeptidase inhibitor activity |
| BB160016B20D09 | CG3019 | FBgn0050197 | su(w[a]) suppressor of white-apricot | GO:0000398 | Bio. Process | nuclear mRNA splicing, via spliceosome |
|  |  |  |  | GO:0005634 | Cell. Component | nucleus |
|  |  |  |  | GO:0005681 | Cell. Component | spliceosome |
| BB160016B20E04 | CG18389 | FBgn0013948 | Eip93F | GO:0048102 | Bio. Process | autophagic cell death |
|  |  |  |  | GO:0006914 | Bio. Process | autophagy |
|  |  |  |  | GO:0035072 | Bio. Process | ecdysone-mediated induction of salivary gland cell autophagic cell death |
|  |  |  |  | GO:0008628 | Bio. Process | induction of apoptosis by hormones |
|  |  |  |  | GO:0035069 | Bio. Process | larval midgut histolysis |
|  |  |  |  | GO:0006911 | Bio. Process | phagocytosis, engulfment |
|  |  |  |  | GO:0045893 | Bio. Process | positive regulation of transcription, DNA-dependent |
|  |  |  |  | GO:0045449 | Bio. Process | regulation of transcription |
|  |  |  |  | GO:0035071 | Bio. Process | salivary gland cell autophagic cell death |
|  |  |  |  | GO:0005634 | Cell. Component | nucleus |
|  |  |  |  | GO:0005700 | Cell. Component | polytene chromosome |
|  |  |  |  | GO:0003677 | Mol. Function | DNA binding |
|  |  |  |  | GO:0003700 | Mol. Function | transcription factor activity |
| BB160017B20C02 | CG1101 | FBgn0010774 | Aly | GO:0031965 | Cell. Component | muclear membrane |
|  |  |  |  | GO:0005654 | Cell. Component | nucleoplasm |
|  |  |  |  | GO:0003729 | Mol. Function | mRNA binding |
|  |  |  |  | GO:0003713 | Mol. Function | transcription cofactor activity |
| BB160019A10H11 | CG31022 | FBgn0039776 | PH4alphaEFB prolyl-4-hydroxylase-alpha EFB | GO:0018401 | Bio. Process | peptidyl-proline hydroxylation to 4-hydroxy-L-proline |
|  |  |  |  | GO:0016222 | Cell. Component | procollagen-proline, 2-oxoglutarate-4-dioxygenase complex |
|  |  |  |  | GO:0004656 | Mol. Function | procollagen-proline 4-dioxygenase activity |
| BB160019A20F11 | CG33097 | FBgn0053097 |  | GO:0006354 | Bio. Process | RNA elongation |
|  |  |  |  | GO:0005634 | Cell. Component | nucleus |
|  |  |  |  | GO:0003700 | Mol. Function | transcription factor activity |
| BB160019A20F12 | CG8967 | FBgn0004839 | otk off-track | GO:0007411 | Bio. Process | axon guidance |
|  |  |  |  | GO:0007155 | Bio. Process | cell adhesion |
|  |  |  |  | GO:0006468 | Bio. Process | protein amino acid phosphorylation |
|  |  |  |  | GO:0005886 | Cell. Component | plasma membrane |
|  |  |  |  | GO:0050839 | Mol. Function | cell adhesion molecule binding |
|  |  |  |  | GO:0004672 | Mol. Function | protein kinase activity |
|  |  |  |  | GO:0004713 | Mol. Function | protein tyrosine kinase activity |
|  |  |  |  | GO:0004872 | Mol. Function | receptor activity |
|  |  |  |  | GO:0030215 | Mol. Function | semaphorin receptor binding |
|  |  |  |  | GO:0004714 | Mol. Function | transmembrane receptor protein tyrosine kinase activity |
| BB160019B10E07 | CG7740 | FBgn0026189 | prominin-like | GO:0005886 | Cell. Component | plasma membrane |
| BB160019B10H06 | CG6772 | FBgn0024290 | Slob Slowpoke binding protein | GO:0050804 | Bio. Process | regulation of synaptic transmission |
|  |  |  |  | GO:0005737 | Cell. Component | cytoplasm |
|  |  |  |  | GO:0005515 | Mol. Function | protein binding |
|  |  |  |  | GO:0004672 | Mol. Function | protein kinase activity |
| BB160019B20C01 | CG4143 | FBgn0026208 | mbf1 multiprotein bridging factor 1 | GO:0007417 | Bio. Process | central nervous system development |
|  |  |  |  | GO:0048813 | Bio. Process | dendrite morphogenesis |
|  |  |  |  | GO:0007424 | Bio. Process | open tracheal system development |
|  |  |  |  | GO:0005737 | Cell. Component | cytoplasm |
|  |  |  |  | GO:0005634 | Cell. Component | nucleus |
|  |  |  |  | GO:0008327 | Mol. Function | methyl-CpG binding |
|  |  |  |  | GO:0003713 | Mol. Function | transcription cofactor activity |
| BB160020A10H01 | CG4357 | FBgn0036279 | Ncc69 sodium chloride cotransporter 69 | GO:0015378 | Mol. Function | sodium:chloride symporter activity |
| BB160021A10E07 | CG31140 | FBgn0051140 |  | GO:0016310 | Bio. Process | phosphorylation |
|  |  |  |  | GO:0019992 | Mol. Function | diacylglycerol binding |
|  |  |  |  | GO:0004143 | Mol. Function | diacylglycerol kinase activity |
| BB160021A10F10 | CG8384 | FBgn0001139 | gro groucho | GO:0007015 | Bio. Process | actin filament organization |
|  |  |  |  | GO:0048813 | Bio. Process | dendrite morphogenesis |
|  |  |  |  | GO:0016481 | Bio. Process | negative regulation of transcription |
|  |  |  |  | GO:0000122 | Bio. Process | negative regulation of transcription from RNA polymerase II promoter |
|  |  |  |  | GO:0030178 | Bio. Process | negative regulation of Wnt receptor signaling pathway |
|  |  |  |  | GO:0007399 | Bio. Process | nervous system development |
|  |  |  |  | GO:0007541 | Bio. Process | sex determination, primary response to X:A ratio |
|  |  |  |  | GO:0005634 | Cell. Component | nucleus |
|  |  |  |  | GO:0003677 | Mol. Function | DNA binding |
|  |  |  |  | GO:0005515 | Mol. Function | protein binding |
|  |  |  |  | GO:0003714 | Mol. Function | transcription corepressor activity |
| BB160021B20C04 | CG4690 | FBgn0029837 | Tsp5D Tetraspanin 5D | GO:0016021 | Cell. Component | integral to membrane |
| BB160021B20C06 | CG4241 | FBgn0067783 | att-ORFA alternative testis transcripts open reading frame A | GO:0005740 | Cell. Component | mitochondrial envelope |
|  |  |  |  | GO:0022857 | Mol. Function | transmembrane transporter activity |
| BB160021B20E02 | CG6303 | FBgn0037808 | Bruce | GO:0006916 | Bio. Process | anti=apoptosis |
|  |  |  |  | GO:0012501 | Bio. Process | programmed cell death |
|  |  |  |  | GO:0007291 | Bio. Process | sperm individualization |
|  |  |  |  | GO:0007286 | Bio. Process | spermatid development |
|  |  |  |  | GO:0019787 | Mol. Function | small conjugating protein ligase activity |
|  |  |  |  | GO:0004842 | Mol. Function | ubiquitin-protein ligase activity |
| BB160021B20H07 | CG1630 | FBgn0030471 | IP3K2 Inositol 1,4,5-triphosphate kinase 2 | GO:0046853 | Bio. Process | inositol and derivative phosphorylation |
|  |  |  |  | GO:0048306 | Mol. Function | calcium-dependent protein binding |
|  |  |  |  | GO:0005516 | Mol. Function | calmodulin binding |
|  |  |  |  | GO:0008440 | Mol. Function | inositol trisphosphate 3-kinase activity |
| BB160022A20C04 | CG16944 | FBgn0003360 | sesB stress-sensitive B | GO:0015866 | Bio. Process | ADP transport |
|  |  |  |  | GO:0015867 | Bio. Process | ATP transport |
|  |  |  |  | GO:0007629 | Bio. Process | flight behavior |
|  |  |  |  | GO:0009612 | Bio. Process | response to mechanical stimulus |
|  |  |  |  | GO:0016021 | Cell. Component | integral to membrane |
|  |  |  |  | GO:0005811 | Cell. Component | lipid particle |
|  |  |  |  | GO:0005740 | Cell. Component | mitochondrial envelope |
|  |  |  |  | GO:0005743 | Cell. Component | mitochondrial inner membrane |
|  |  |  |  | GO:0005471 | Mol. Function | ATP:ADP antiporter activity |
|  |  |  |  | GO:0022857 | Mol. Function | transmembrane transporter actvity |
| BB160022A20D04 | CG3421 | FBgn0038853 | RhoGAP93B | GO:0007411 | Bio. Process | axon guidance |
| BB160022A20G06 | CG5001 | FBgn0031322 |  | GO:0009408 | Bio. Process | response to heat |
| BB160022A20H05 | CG5627 | FBgn0030613 | rab3-GEF | GO:0000187 | Bio. Process | activation of MAPK activity |
|  |  |  |  | GO:0007269 | Bio. Process | neurotransmitter secretion |
|  |  |  |  | GO:0042981 | Bio. Process | regulation of apoptosis |
|  |  |  |  | GO:0051726 | Bio. Process | regulation of cell cycle |
|  |  |  |  | GO:0016192 | Bio. Process | vesicle-mediated transport |
|  |  |  |  | GO:0016021 | Cell. Component | integral to membrane |
|  |  |  |  | GO:0008021 | Cell. Component | synaptic vesicle |
|  |  |  |  | GO:0017112 | Mol. Function | Rab guanyl-nucleotide exchange factor activity |
| BB160022B20G08 | CG4257 | FBgn0016917 | Stat92E Signal-transducer and activator of transcription protein at 92E | GO:0007350 | Bio. Process | blastoderm segmentation |
|  |  |  |  | GO:0007298 | Bio. Process | border follicle cell migration |
|  |  |  |  | GO:0048749 | Bio. Process | compound eye development |
|  |  |  |  | GO:0006952 | Bio. Process | defense response |
|  |  |  |  | GO:0007425 | Bio. Process | epithelial cell fate determination, open tracheal system |
|  |  |  |  | GO:0007455 | Bio. Process | eye-antennal disc |
|  |  |  |  | GO:0042078 | Bio. Process | germ-line stem cell division |
|  |  |  |  | GO:0030718 | Bio. Process | germ-line stem cell maintenance |
|  |  |  |  | GO:0035172 | Bio. Process | hemocyte proliferation |
|  |  |  |  | GO:0030097 | Bio. Process | hemopoiesis |
|  |  |  |  | GO:0007442 | Bio. Process | hindgut morphogenesis |
|  |  |  |  | GO:0006059 | Bio. Process | humoral immune response |
|  |  |  |  | GO:0007476 | Bio. Process | imaginal disc-derived wing |
|  |  |  |  | GO:0007259 | Bio. Process | JAK-STAT cascade |
|  |  |  |  | GO:0035171 | Bio. Process | lamellocyte differentiation |
|  |  |  |  | GO:0030178 | Bio. Process | negative regulation of Wnt receptor signaling pathway |
|  |  |  |  | GO:0007399 | Bio. Process | nervous system development |
|  |  |  |  | GO:0030720 | Bio. Process | oocyte localization during germarium-derived egg chamber formation |
|  |  |  |  | GO:0048477 | Bio. Process | oogenesis |
|  |  |  |  | GO:0007424 | Bio. Process | open tracheal system development |
|  |  |  |  | GO:0030713 | Bio. Process | ovarian follicle cell stalk formation |
|  |  |  |  | GO:0007538 | Bio. Process | primary sex determination |
|  |  |  |  | GO:0045449 | Bio. Process | regulation of transcription |
|  |  |  |  | GO:0007379 | Bio. Process | segment specification |
|  |  |  |  | GO:0007530 | Bio. Process | sex determination |
|  |  |  |  | GO:0007165 | Bio. Process | signal transduction |
|  |  |  |  | GO:0048103 | Bio. Process | somatic stem cell division |
|  |  |  |  | GO:0017145 | Bio. Process | stem cell division |
|  |  |  |  | GO:0019827 | Bio. Process | stem cell maintenance |
|  |  |  |  | GO:0005737 | Cell. Component | cytoplasm |
|  |  |  |  | GO:0005634 | Cell. Component | nucleus |
|  |  |  |  | GO:0003677 | Mol. Function | DNA binding |
|  |  |  |  | GO:0005515 | Mol. Function | protein binding |
|  |  |  |  | GO:0003702 | Mol. Function | RNA polymerase II transcription factor activity |
|  |  |  |  | GO:0003700 | Mol. Function | transcription factor activity |
| BB160023A10B09 | CG15797 | FBgn0028292 | ric8a | GO:0008356 | Bio. Process | asymmetric cell division |
|  |  |  |  | GO:0055059 | Bio. Process | asymmetric neuroblast division |
|  |  |  |  | GO:0008105 | Bio. Process | asymmetric protein localization |
|  |  |  |  | GO:0007163 | Bio. Process | establishment and/or maintenance of cell polarity |
|  |  |  |  | GO:0040001 | Bio. Process | establishment of mitotic spindle localization |
|  |  |  |  | GO:0010004 | Bio. Process | gastrulation involving germ band extension |
|  |  |  |  | GO:0050821 | Bio. Process | protein stabilization |
|  |  |  |  | GO:0005737 | Cell. Component | cytoplasm |
|  |  |  |  | GO:0005829 | Cell. Component | cytosol |
|  |  |  |  | GO:0001965 | Mol. Function | G-protein alpha-subunit binding |
|  |  |  |  | GO:0005085 | Mol. Function | guanyl-nucleotide exchange factor activity |
| BB160023B10A01 | CG8543 | FBgn0035787 |  | GO:0005214 | Mol. Function | structural constituent of chitin-based cuticle |
| BB160023B10C08 | CG5889 | FBgn0029155 | Mdh | GO:0006108 | Bio. Process | malate metabolic process |
|  |  |  |  | GO:0006090 | Bio. Process | pyruvate metabolic process |
|  |  |  |  | GO:0005739 | Cell. Component | mitochondrion |
|  |  |  |  | GO:0004473 | Mol. Function | malate dehydrogenase (oxaloacetate-decarboxylating) (NADP+) activity |
| BB160024A10A12 | CG12598 | FBgn0026086 | Adar Adenosine deaminase acting on RNA | GO:0006382 | Bio. Process | adenosine to inosine editing |
|  |  |  |  | GO:0030534 | Bio. Process | adult behavior |
|  |  |  |  | GO:0008344 | Bio. Process | adult locomotory behavior |
|  |  |  |  | GO:0007626 | Bio. Process | locomotory behavior |
|  |  |  |  | GO:0016556 | Bio. Process | mRNA modification |
|  |  |  |  | GO:0042391 | Bio. Process | regulation of membrane potential |
|  |  |  |  | GO:0009408 | Bio. Process | response to heat |
|  |  |  |  | GO:0001666 | Bio. Process | response to hypoxia |
|  |  |  |  | GO:0003726 | Mol. Function | double-stranded RNA adenosine deaminase activity |
|  |  |  |  | GO:0003725 | Mol. Function | double-stranded RNA binding |
| BB160024A20C03 | CG6331 | FBgn0019952 | Orct Organic cation transporter | GO:0006915 | Bio. Process | apoptosis |
|  |  |  |  | GO:0015695 | Bio. Process | organic cation transport |
|  |  |  |  | GO:0016021 | Cell. Component | integral to membrane |
|  |  |  |  | GO:0015101 | Mol. Function | organic cation transmembrane transporter activity |
| BB160024A20C06 | CG8646 | FBgn0033763 |  | GO:0003943 | Mol. Function | N-acetylgalactosamine-4-sulfatase activity |
| BB160024B20F01 | CG5889 | FBgn0029155 | Mdh | GO:0006108 | Bio. Process | malate metabolic process |
|  |  |  |  | GO:0006090 | Bio. Process | pyruvate metabolic process |
|  |  |  |  | GO:0005739 | Cell. Component | mitochondrion |
|  |  |  |  | GO:0004473 | Mol. Function | malate dehydrogenase (oxaloacetate-decarboxylating) (NADP+) activity |
| BB170001B10H02 | CG7057 | FBgn0024832 | AP-50 | GO:0007269 | Bio. Process | neurotransmitter secretion |
|  |  |  |  | GO:0016183 | Bio. Process | synaptic vesicle coating |
|  |  |  |  | GO:0006901 | Bio. Process | vesicle coating |
|  |  |  |  | GO:0030135 | Cell. Component | coated vesicle |
|  |  |  |  | GO:0008021 | Cell. Component | synaptic vesicle |
| BB170001B10H06 | CG5760 | FBgn0028468 | rtet tetracycline resistance | GO:0048477 | Bio. Process | oogenesis |
|  |  |  |  | GO:0051119 | Mol. Function | sugar transmembrane transporter activity |
| BB170002A10B10 | CG5119 | FBgn0003031 | pAbp polyA-binding protein | GO:0045727 | Bio. Process | positive regulation of translation |
|  |  |  |  | GO:0007268 | Bio. Process | synaptic transmission |
|  |  |  |  | GO:0005737 | Cell. Component | cytoplasm |
|  |  |  |  | GO:0005811 | Cell. Component | lipid particle |
|  |  |  |  | GO:0003729 | Mol. Function | mRNA binding |
|  |  |  |  | GO:0008143 | Mol. Function | poly(A) binding |
|  |  |  |  | GO:0005515 | Mol. Function | protein binding |
| BB170002A10G12 | CG32387 | FBgn0052387 |  | GO:0007156 | Bio. Process | homophilic cell adhesion |
| BB170002B10G12 | CG4382 | FBgn0032132 |  | GO:0004091 | Mol. Function | carboxylesterase activity |
| BB170002B10H05 | CG6303 | FBgn0037808 | Bruce | GO:0006916 | Bio. Process | anti=apoptosis |
|  |  |  |  | GO:0012501 | Bio. Process | programmed cell death |
|  |  |  |  | GO:0007291 | Bio. Process | sperm individualization |
|  |  |  |  | GO:0007286 | Bio. Process | spermatid development |
|  |  |  |  | GO:0019787 | Mol. Function | small conjugating protein ligase activity |
|  |  |  |  | GO:0004842 | Mol. Function | ubiquitin-protein ligase activity |
| BB170002B20H06 | CG4032 | FBgn0000017 | Abl Abl tyrosine kinase | GO:0007411 | Bio. Process | axon guidance |
|  |  |  |  | GO:0007417 | Bio. Process | central nervous system development |
|  |  |  |  | GO:0007391 | Bio. Process | dorsal closure |
|  |  |  |  | GO:0002009 | Bio. Process | morphogenesis of an epithelium |
|  |  |  |  | GO:0006468 | Bio. Process | protein amino acid phosphorylation |
|  |  |  |  | GO:0008064 | Bio. Process | regulation of actin polymerization and/or depolymerization |
|  |  |  |  | GO:0008360 | Bio. Process | regulation of cell shape |
|  |  |  |  | GO:0007370 | Bio. Process | ventral furrow formation |
|  |  |  |  | GO:0005912 | Cell. Component | adherens junction |
|  |  |  |  | GO:0045179 | Cell. Component | apical cortex |
|  |  |  |  | GO:0005737 | Cell. Component | cytoplasm |
|  |  |  |  | GO:0019897 | Cell. Component | extrinsic to plasma membrane |
|  |  |  |  | GO:0005911 | Cell. Component | intercellular junction |
|  |  |  |  | GO:0005927 | Cell. Component | muscle tendon junction |
|  |  |  |  | GO:0005524 | Mol. Function | ATP binding |
|  |  |  |  | GO:0004715 | Mol. Function | non-membrane spanning protein tyrosine kinase activity |
|  |  |  |  | GO:0005515 | Mol. Function | protein binding |
|  |  |  |  | GO:0004713 | Mol. Function | protein tyrosine kinase activity |
| BB170003B20C05 | CG1362 | FBgn0039209 | cdc2rk cdc2-related-kinase | GO:0006468 | Bio. Process | protein amino acid phosphorylation |
|  |  |  |  | GO:0004693 | Mol. Function | cyclin-dependent protein kinase activity |
|  |  |  |  | GO:0004674 | Mol. Function | protein serine/threonine kinase activity |
| BB170006A10B12 | CG32180 | FBgn0000567 | Eip74EF Ecdysone-induced protein 74EF | GO:0006914 | Bio. Process | autophagy |
|  |  |  |  | GO:0008219 | Bio. Process | cell death |
|  |  |  |  | GO:0009987 | Bio. Process | cellular process |
|  |  |  |  | GO:0048477 | Bio. Process | oogenesis |
|  |  |  |  | GO:0040034 | Bio. Process | regulation of development, heterochronic |
|  |  |  |  | GO:0045449 | Bio. Process | regulation of transcription |
|  |  |  |  | GO:0006355 | Bio. Process | regulation of transcription, DNA-dependent |
|  |  |  |  | GO:0035071 | Bio. Process | salivary gland cell autophagic cell death |
|  |  |  |  | GO:0005634 | Cell. Component | nucleus |
|  |  |  |  | GO:0003704 | Mol. Function | specific RNA polymerase II transcription factor activity |
|  |  |  |  | GO:0003700 | Mol. Function | transcription factor activity |
| BB170006A10E10 | CG8582 | FBgn0035772 | Sh3beta | GO:0005575 | Cell. Component | cellular_component |
| BB170007A20B12 | CG32121 | FBgn0052121 |  | GO:0006357 | Bio. Process | regulation of transcription from RNA polymerase II promoter |
|  |  |  |  | GO:0003700 | Mol. Function | transcription factor activity |
| BB170007B10A02 | CG15002 | FBgn0011653 | mas masquerade | GO:0005886 | Cell. Component | plasma membrane |
|  |  |  |  | GO:0008233 | Mol. Function | peptidase activity |
|  |  |  |  | GO:0004252 | Mol. Function | serine-type endopeptidase activity |
| BB170008A10G05 | CG1973 | FBgn0039692 |  | GO:0004672 | Mol. Function | protein kinase activity |
| BB170008A20H01 | CG8808 | FBgn0017558 | Pdk Pyruvate dehydrogenase kinase | GO:0006090 | Bio. Process | pyruvate metabolic process |
|  |  |  |  | GO:0005759 | Cell. Component | mitochondrial matrix |
|  |  |  |  | GO:0004740 | Mol. Function | pyruvate dehydrogenase (acetyl-transferring) kinase activity |
| BB170008B10G08 | CG7430 | FBgn0036762 |  | GO:0006546 | Bio. Process | glycine catabolic process |
|  |  |  |  | GO:0006748 | Bio. Process | lipoamide metabolic process |
|  |  |  |  | GO:0006099 | Bio. Process | tricarboxylic acid cycle |
|  |  |  |  | GO:0005960 | Cell. Component | glycine cleavage complex |
|  |  |  |  | GO:0005811 | Cell. Component | lipid particle |
|  |  |  |  | GO:0005947 | Cell. Component | mitochondrial alpha-ketoglutarate dehydrogenase complex |
|  |  |  |  | GO:0004148 | Mol. Function | dihydrolipoyl dehydrogenase activity |
| BB170010A10G11 | CG10360 | FBgn0003231 | ref(2)P refractory to sigma P | GO:0019058 | Bio. Process | viral infectious cycle |
|  |  |  |  | GO:0005634 | Cell. Component | nucleus |
| BB170010B10H01 | CG2926 | FBgn0037344 |  | GO:0000381 | Bio. Process | regulation of alternative nuclear mRNA splicing, via spliceosome |
|  |  |  |  | GO:0005634 | Cell. Component | nucleus |
| BB170010B20B02 | CG13425 | FBgn0015907 | bl bancal | GO:0035107 | Bio. Process | appendage morphogenesis |
|  |  |  |  | GO:0045165 | Bio. Process | cell fate commitment |
|  |  |  |  | GO:0008283 | Bio. Process | cell proliferation |
|  |  |  |  | GO:0007446 | Bio. Process | imaginal disc growth |
|  |  |  |  | GO:0000381 | Bio. Process | regulation of alternative nuclear mRNA splicing, via spliceosome |
|  |  |  |  | GO:0000785 | Cell. Component | chromatin |
|  |  |  |  | GO:0005737 | Cell. Component | cytoplasm |
|  |  |  |  | GO:0005634 | Cell. Component | nucleus |
|  |  |  |  | GO:0035062 | Cell. Component | omega speckle |
|  |  |  |  | GO:0030529 | Cell. Component | ribonucleoprotein complex |
|  |  |  |  | GO:0003729 | Mol. Function | mRNA binding |
|  |  |  |  | GO:0008134 | Mol. Function | transcription factor binding |
| BB170011A10D10 | CG32593 | FBgn0024753 | Flo-2 flotillin 2 | GO:0007155 | Bio. Process | cell adhesion |
|  |  |  |  | GO:0016600 | Cell. Component | flotillin complex |
|  |  |  |  | GO:0005198 | Mol. Function | structural molecule activity |
| BB170011A20H07 | CG12013 | FBgn0035438 | PHGPx | GO:0006982 | Bio. Process | response to lipid hydroperoxide |
|  |  |  |  | GO:0005737 | Cell. Component | cytoplasm |
|  |  |  |  | GO:0005739 | Cell. Component | mitochondrion |
|  |  |  |  | GO:0004602 | Mol. Function | glutathion peroxidase activity |
|  |  |  |  | GO:0004601 | Mol. Function | peroxidase activity |
| BB170011B10H10 | CG1507 | FBgn0022361 | Pur-alpha Purine-rich binding protein-alpha | GO:0003697 | Mol. Function | single-stranded DNA binding |
|  |  |  |  | GO:0016563 | Mol. Function | transcription activator activity |
| BB170012A10D08 | CG12806 | FBgn0037766 | Teh1 tipE homolog 1 | GO:0016974 | Mol. Function | sodium channel auxiliary protein activity |
| BB170012B10G06 | CG3727 | FBgn0010583 | dock dreadlocks | GO:0007411 | Bio. Process | axon guidance |
|  |  |  |  | GO:0007409 | Bio. Process | axonogenesis |
|  |  |  |  | GO:0008286 | Bio. Process | insulin receptor signaling pathway |
|  |  |  |  | GO:0005737 | Cell. Component | cytoplasm |
|  |  |  |  | GO:0005158 | Mol. Function | insulin receptor binding |
|  |  |  |  | GO:0005070 | Mol. Function | SH3/SH2 adaptor |
| BB170012B20B03 | CG17759 | FBgn0004435 | Galpha49B G protein alpha49B | GO:0007202 | Bio. Process | acdtivation of phospholipase C activity |
|  |  |  |  | GO:0016199 | Bio. Process | axon midline choice point recognition |
|  |  |  |  | GO:0007186 | Bio. Process | G-protein coupled receptor protein signaling pathway |
|  |  |  |  | GO:0046673 | Bio. Process | negative regulation of compound eye retinal cell programmed cell death |
|  |  |  |  | GO:0007602 | Bio. Process | phototransduction |
|  |  |  |  | GO:0016056 | Bio. Process | rhodopsin mediated signaling pathway |
|  |  |  |  | GO:0005834 | Cell. Component | heterotrimeric G-protein complex |
|  |  |  |  | GO:0016027 | Cell. Component | inaD signaling complex |
|  |  |  |  | GO:0016028 | Cell. Component | rhabdomere |
|  |  |  |  | GO:0003924 | Mol. Function | GTPase activity |
| BB170013A10H04 | CG6476 | FBgn0003600 | Su(var)3-9 Suppressor of variegation 3-9 | GO:0006342 | Bio. Process | chromatin silencing |
|  |  |  |  | GO:0030702 | Bio. Process | chromatin silencing at centromere |
|  |  |  |  | GO:0006348 | Bio. Process | chromatin silencing at telomere |
|  |  |  |  | GO:0051276 | Bio. Process | chromosome organization and biogenesis |
|  |  |  |  | GO:0006306 | Bio. Process | DNA methylation |
|  |  |  |  | GO:0006325 | Bio. Process | establishment and/or maintenance of chromatin architecture |
|  |  |  |  | GO:0016458 | Bio. Process | gene silencing |
|  |  |  |  | GO:0051567 | Bio. Process | histone H3-K9 methylation |
|  |  |  |  | GO:0016571 | Bio. Process | histone methylation |
|  |  |  |  | GO:0016570 | Bio. Process | histone modification |
|  |  |  |  | GO:0048477 | Bio. Process | oogenesis |
|  |  |  |  | GO:0006413 | Bio. Process | translational initiation |
|  |  |  |  | GO:0000775 | Cell. Component | chromosome, centromeric region |
|  |  |  |  | GO:0005829 | Cell. Component | cytosol |
|  |  |  |  | GO:0005850 | Cell. Component | eukaryotic translation initiation facor 2 complex |
|  |  |  |  | GO:0000792 | Cell. Component | heterochromatin |
|  |  |  |  | GO:0005634 | Cell. Component | nucleus |
|  |  |  |  | GO:0003682 | Mol. Function | chromatin binding |
|  |  |  |  | GO:0005525 | Mol. Function | GTP binding |
|  |  |  |  | GO:0046974 | Mol. Function | histone lysine N-methyltransferase activity (H3-K9 specific) |
|  |  |  |  | GO:0042054 | Mol. Function | histone methyltransferase activity |
|  |  |  |  | GO:0003743 | Mol. Function | translation initiation factor activity |
|  |  |  |  | GO:0000049 | Mol. Function | tRNA binding |
| BB170013A20H08 | CG10443 | FBgn0000464 | Lar Leukocyte-antigen-related-like | GO:0007411 | Bio. Process | axon guidance |
|  |  |  |  | GO:0007155 | Bio. Process | cell adhesion |
|  |  |  |  | GO:0001700 | Bio. Process | embryonic development via the syncytial blastoderm |
|  |  |  |  | GO:0008045 | Bio. Process | motor axon development |
|  |  |  |  | GO:0007399 | Bio. Process | nervous system development |
|  |  |  |  | GO:0048477 | Bio. Process | oogenesis |
|  |  |  |  | GO:0008594 | Bio. Process | photoreceptor cell morphogenesis |
|  |  |  |  | GO:0006470 | Bio. Process | protein amino acid dephosphorylation |
|  |  |  |  | GO:0045467 | Bio. Process | R7 cell development |
|  |  |  |  | GO:0008360 | Bio. Process | regulation of cell shape |
|  |  |  |  | GO:0031290 | Bio. Process | retinal ganglion cell axon guidance |
|  |  |  |  | GO:0016021 | Cell. Component | integral to membrane |
|  |  |  |  | GO:0005886 | Cell. Component | plasma membrane |
|  |  |  |  | GO:0004725 | Mol. Function | protein tyrosine phosphatase activity |
|  |  |  |  | GO:0005001 | Mol. Function | transmembrane receptor protein tyrosine phosphatase activity |
| BB170013B10E11 | CG3394 | FBgn0034999 |  | GO:0005324 | Mol. Function | long-chain fatty acid transporter |
| BB170013B20C06 | CG5269 | FBgn0026158 | vib vibrator | GO:0035091 | Mol. Function | phosphoinositide binding |
|  |  |  |  | GO:0005548 | Mol. Function | phospholipid transporter activity |
| BB170014B10F06 | CG8171 | FBgn0000996 | dup double parked | GO:0019730 | Bio. Process | antimicrobial humoral response |
|  |  |  |  | GO:0007304 | Bio. Process | chorion-containing eggshell formation |
|  |  |  |  | GO:0006260 | Bio. Process | DNA replication |
|  |  |  |  | GO:0000076 | Bio. Process | DNA replication checkpoint |
|  |  |  |  | GO:0006261 | Bio. Process | DNA-dependent DNA replication |
|  |  |  |  | GO:0007307 | Bio. Process | eggshell chorion gene amplification |
|  |  |  |  | GO:0005737 | Cell. Component | cytoplasm |
|  |  |  |  | GO:0005664 | Cell. Component | nuclear origin of replication recognition complex |
|  |  |  |  | GO:0005634 | Cell. Component | nucleus |
|  |  |  |  | GO:0005657 | Cell. Component | replication fork |
|  |  |  |  | GO:0003677 | Mol. Function | DNA binding |
| BB170015B10D04 | CG7199 | FBgn0015239 | Hr78 Hormone-receptor-like in 78 | GO:0048102 | Bio. Process | autophagic cell death |
|  |  |  |  | GO:0035002 | Bio. Process | liquid clearance, open tracheal system |
|  |  |  |  | GO:0007424 | Bio. Process | open tracheal system development |
|  |  |  |  | GO:0035071 | Bio. Process | salivary gland cell autophagic cell death |
|  |  |  |  | GO:0005634 | Cell. Component | nucleus |
|  |  |  |  | GO:0003677 | Mol. Function | DNA binding |
|  |  |  |  | GO:0004879 | Mol. Function | ligand-dependent nuclear receptor activity |
|  |  |  |  | GO:0003700 | Mol. Function | transcription factor activity |
| BB170015B20G12 | CG8250 | FBgn0040505 | Alk | GO:0000186 | Bio. Process | activation of MAPKK activity |
|  |  |  |  | GO:0007411 | Bio. Process | axon guidance |
|  |  |  |  | GO:0042051 | Bio. Process | compound eye photoreceptor |
|  |  |  |  | GO:0048565 | Bio. Process | gut development |
|  |  |  |  | GO:0007498 | Bio. Process | mesoderm development |
|  |  |  |  | GO:0006468 | Bio. Process | protein amino acid phosphorylation |
|  |  |  |  | GO:0045610 | Bio. Process | regulation of hemocyte differentiation |
|  |  |  |  | GO:0007522 | Bio. Process | vesceral muscle development |
|  |  |  |  | GO:0005886 | Cell. Component | plasma membrane |
|  |  |  |  | GO:0004713 | Mol. Function | protein tyrosine kinase activity |
|  |  |  |  | GO:0004716 | Mol. Function | receptor signaling protein tyrosine kinase acitivty |
|  |  |  |  | GO:0004714 | Mol. Function | transmembrane receptor protein tyrosine kinase activity |
| BB170016A10E11 | CG33526 | FBgn0053526 | PNUTS | GO:0006911 | Bio. Process | phagocytosis, engulfment |
|  |  |  |  | GO:0006605 | Bio. Process | protein targeting |
|  |  |  |  | GO:0050790 | Bio. Process | regulation of catalytic activity |
|  |  |  |  | GO:0035304 | Bio. Process | regulation of protein amino acid dephosphorylation |
|  |  |  |  | GO:0005634 | Cell. Component | nucleus |
|  |  |  |  | GO:0019888 | Mol. Function | protein phosphatase regulator activity |
| BB170016B10A03 | CG4590 | FBgn0027108 | inx2 innexin 2 | GO:0007440 | Bio. Process | foregut morphogenesis |
|  |  |  |  | GO:00162331 | Bio. Process | morphogenesis of embryonic epithelium |
|  |  |  |  | GO:0042048 | Bio. Process | olfactory behavior |
|  |  |  |  | GO:0005921 | Cell. Component | gap junction |
|  |  |  |  | GO:0016021 | Cell. Component | integral to membrane |
|  |  |  |  | GO:0005243 | Mol. Function | gap junction channel activity |
| BB170016B10B03 | CG1448 | FBgn0034245 | inx3 innexin 3 | GO:0005921 | Cell. Component | gap junction |
|  |  |  |  | GO:0016021 | Cell. Component | integral to membrane |
|  |  |  |  | GO:0005243 | Mol. Function | gap junction channel activity |
| BB170016B10F05 | CG2092 | FBgn0004243 | scra scraps | GO:0007349 | Bio. Process | cellularization |
|  |  |  |  | GO:0000910 | Bio. Process | cytokinesis |
|  |  |  |  | GO:0007009 | Bio. Process | plasma membrane organization and biogenesis |
|  |  |  |  | GO:0031106 | Bio. Process | septin ring organization |
|  |  |  |  | GO:0005826 | Cell. Component | contractile ring |
|  |  |  |  | GO:0005737 | Cell. Component | cytoplasm |
|  |  |  |  | GO:0045172 | Cell. Component | germline ring canal |
|  |  |  |  | GO:0005634 | Cell. Component | nucleus |
|  |  |  |  | GO:0003779 | Mol. Function | actin binding |
|  |  |  |  | GO:0008017 | Mol. Function | microtubule binding |
| BB170017A10C02 | CG5413 | FBgn0025456 | CREG Cellular Repressor of E1A-stimulated genes | GO:0016481 | Bio. Process | negative regulation of transcription |
|  |  |  |  | GO:0005634 | Cell. Component | nucleus |
|  |  |  |  | GO:0005515 | Mol. Function | protein binding |
|  |  |  |  | GO:0016564 | Mol. Function | transcription repressor activity |
| BB170017A10D12 | CG33991 | FBgn0013718 | nuf nuclear fallout | GO:0031532 | Bio. Process | actin cytoskeleton reorganization |
|  |  |  |  | GO:0000915 | Bio. Process | cytokinesis, contractile ring formation |
|  |  |  |  | GO:0007017 | Bio. Process | microtubule-based process |
|  |  |  |  | GO:0016360 | Bio. Process | sensory organ precursor cell fate determination |
|  |  |  |  | GO:0005813 | Cell. Component | centrosome |
|  |  |  |  | GO:0005875 | Cell. Component | microtubule associated complex |
|  |  |  |  | GO:0008017 | Mol. Function | microtubule binding |
| BB170017A10D12 | CG7867 | FBgn0013718 | nuf nuclear fallout | GO:0031532 | Bio. Process | actin cytoskeleton reorganization |
|  |  |  |  | GO:0000915 | Bio. Process | cytokinesis, contractile ring formation |
|  |  |  |  | GO:0007017 | Bio. Process | microtubule-based process |
|  |  |  |  | GO:0016360 | Bio. Process | sensory organ precursor cell fate determination |
|  |  |  |  | GO:0005813 | Cell. Component | centrosome |
|  |  |  |  | GO:0005875 | Cell. Component | microtubule associated complex |
|  |  |  |  | GO:0008017 | Mol. Function | microtubule binding |
| BB170017A20D11 | CG5905 | FBgn0029843 | Nep1 Neprilysin 1 | GO:0004222 | Mol. Function | metalloendopeptidase activity |
|  |  |  |  | GO:0008237 | Mol. Function | metallopeptidase activity |
| BB170017B10A03 | CG4164 | FBgn0031256 |  | GO:0009408 | Bio. Process | resonse to heat |
| BB170017B20D06 | CG3822 | FBgn0038837 |  | GO:0015277 | Mol. Function | kainate selective glutamate receptor activity |
| BB170018A20C02 | CG6391 | FBgn0036111 | Aps | GO:0008486 | Mol. Function | diphosphoinositol-polyphosphate diphosphatase activity |
| BB170018B10H04 | CG31136 | FBgn0013343 | Syx1A Syntaxin 1A | GO:0007349 | Bio. Process | cellularization |
|  |  |  |  | GO:0042335 | Bio. Process | cuticle development |
|  |  |  |  | GO:0000910 | Bio. Process | cytokinesis |
|  |  |  |  | GO:0006887 | Bio. Process | exocytosis |
|  |  |  |  | GO:0007482 | Bio. Process | haltere development |
|  |  |  |  | GO:0006936 | Bio. Process | muscle contraction |
|  |  |  |  | GO:0007269 | Bio. Process | neurotransmitter secretion |
|  |  |  |  | GO:0007317 | Bio. Process | regulation of pole plasm oskar mRNA localization |
|  |  |  |  | GO:0007268 | Bio. Process | synaptic transmission |
|  |  |  |  | GO:0016081 | Bio. Process | synaptic vesicle docking during exocytosis |
|  |  |  |  | GO:0031629 | Bio. Process | synaptic vesicle fusion to presynaptic membrane |
|  |  |  |  | GO:0016192 | Bio. Process | vesicle-mediated transport |
|  |  |  |  | GO:0016021 | Cell. Component | integral to membrane |
|  |  |  |  | GO:0016020 | Cell. Component | membrane |
|  |  |  |  | GO:0005886 | Cell. Component | plasma membrane |
|  |  |  |  | GO:0005484 | Mol. Function | SNAP receptor activity |
| BB170018B20F10 | CG31671 | FBgn0031390 | tho2 | GO:0006406 | Bio. Process | mRNA export from nucleus |
| BB170019A20F12 | CG10325 | FBgn0000014 | abd-A abdominal A | GO:0006915 | Bio. Process | apoptosis |
|  |  |  |  | GO:0048738 | Bio. Process | cardiac muscle development |
|  |  |  |  | GO:0010002 | Bio. Process | cardioblast differentiation |
|  |  |  |  | GO:0009987 | Bio. Process | cellular process |
|  |  |  |  | GO:0035225 | Bio. Process | determination of genital disc primordium |
|  |  |  |  | GO:0035053 | Bio. Process | dorsal vessel heart proper cell fate commitment |
|  |  |  |  | GO:0035224 | Bio. Process | genital disc anterior/posterior pattern formation |
|  |  |  |  | GO:0048806 | Bio. Process | genitalia development |
|  |  |  |  | GO:0008354 | Bio. Process | germ cell migration |
|  |  |  |  | GO:0008406 | Bio. Process | gonad development |
|  |  |  |  | GO:0007506 | Bio. Process | gonadal mesoderm development |
|  |  |  |  | GO:0007507 | Bio. Process | heart development |
|  |  |  |  | GO:0007501 | Bio. Process | mesodermal cell fate specification |
|  |  |  |  | GO:0007494 | Bio. Process | midgut development |
|  |  |  |  | GO:0007399 | Bio. Process | nervous system development |
|  |  |  |  | GO:0007400 | Bio. Process | neuroblast fate determination |
|  |  |  |  | GO:0007438 | Bio. Process | oenocyte development |
|  |  |  |  | GO:0007422 | Bio. Process | peripheral nervous system development |
|  |  |  |  | GO:0007280 | Bio. Process | pole cell migration |
|  |  |  |  | GO:0007385 | Bio. Process | specification of segmental identity, abdomen |
|  |  |  |  | GO:0005634 | Cell. Component | nucleus |
|  |  |  |  | GO:0003704 | Mol. Function | specific RNA polymerase II transcription factor activity |
| BB170019B20H06 | CG3977 | FBgn0062413 | Ctr1A Copper transporter 1A | GO:0006825 | Bio. Process | copper ion transport |
|  |  |  |  | GO:0005886 | Cell. Component | plasma membrane |
|  |  |  |  | GO:0005375 | Mol. Function | copper ion transmembrane transporter activity |
|  |  |  |  | GO:0015088 | Mol. Function | copper uptake transmembrane transporter activity |
| BB170020A10A03 | CG32490 | FBgn0041605 | cpx complexin | GO:0016079 | Bio. Process | synaptic vesicle exocytosis |
|  |  |  |  | GO:0016020 | Cell. Component | membrane |
|  |  |  |  | GO:0005326 | Mol. Function | neurotransmitter transporter activity |
| BB170020B10C05 | CG6461 | FBgn0030932 |  | GO:0003840 | Mol. Function | gamma-glutamyltransferase activity |
| BB170022A20A04 | CG33106 | FBgn0043884 | mask multiple ankyrin repeats single KH domain | GO:0019730 | Bio. Process | antimicrobial humoral response |
|  |  |  |  | GO:0008283 | Bio. Process | cell proliferation |
|  |  |  |  | GO:0009987 | Bio. Process | cellular process |
|  |  |  |  | GO:0001751 | Bio. Process | compound eye photoreceptor cell differentiation |
|  |  |  |  | GO:0007016 | Bio. Process | cytoskeletal anchoring at plasma membrane |
|  |  |  |  | GO:0007169 | Bio. Process | transmembrane receptor protein tyrosine kinase signaling pathway |
|  |  |  |  | GO:0005811 | Cell. Component | lipid particle |
|  |  |  |  | GO:0005200 | Mol. Function | structural constituent of cytoskeleton |
| BB170022B10B07 | CG5905 | FBgn0029843 | Nep1 Neprilysin 1 | GO:0004222 | Mol. Function | metalloendopeptidase activity |
|  |  |  |  | GO:0008237 | Mol. Function | metallopeptidase activity |
| BB170022B10F05 | CG6984 | FBgn0034191 |  | GO:0004300 | Mol. Function | enoyl-CoA hydratase activity |
| BB170024A10D03 | CG8745 | FBgn0036381 |  | GO:0019544 | Bio. Process | arginine catabolic process |
|  |  |  |  | GO:0005739 | Cell. Component | mitochondrion |
|  |  |  |  | GO:0008453 | Mol. Function | alanine-glyoxylate transaminase activity |
|  |  |  |  | GO:0004587 | Mol. Function | ornithine-oxo-acid transaminase activity |
| BB170024B10F07 | CG9325 | FBgn0004873 | hts hu li tai shao | GO:0051297 | Bio. Process | centrosome organization and biogenesis |
|  |  |  |  | GO:0007282 | Bio. Process | cystoblast division |
|  |  |  |  | GO:0048135 | Bio. Process | female germ-line cyst |
|  |  |  |  | GO:0045478 | Bio. Process | fusome organization and biogenesis |
|  |  |  |  | GO:0048134 | Bio. Process | germ-line cyst formation |
|  |  |  |  | GO:0007294 | Bio. Process | germarium-derived oocyte fate determination |
|  |  |  |  | GO:0000212 | Bio. Process | meiotic spindle organization |
|  |  |  |  | GO:0030723 | Bio. Process | ovarian fusome organization and biogenesis |
|  |  |  |  | GO:0008302 | Bio. Process | ring canal formation, actin assembly |
|  |  |  |  | GO:0030721 | Bio. Process | spectrosome organization and biogenesis |
|  |  |  |  | GO:0030724 | Bio. Process | testicular fusome organization and biogenesis |
|  |  |  |  | GO:0045169 | Cell. Component | fusome |
|  |  |  |  | GO:0045172 | Cell. Component | germline ring canal |
|  |  |  |  | GO:0035183 | Cell. Component | germline ring canal inner rim |
|  |  |  |  | GO:0005811 | Cell. Component | lipid particle |
|  |  |  |  | GO:0005886 | Cell. Component | plasma membrane |
|  |  |  |  | GO:0045170 | Cell. Component | spectrosome |
|  |  |  |  | GO:0003779 | Mol. Function | actin binding |
| BB170024B20C11 | CG7535 | FBgn0024963 | GluClalpha | GO:0008068 | Mol. Function | extracellular-glutamate-gated chloride channel activity |
| BB170025A20C11 | CG32593 | FBgn0024753 | Flo-2 flotillin 2 | GO:0007155 | Bio. Process | cell adhesion |
|  |  |  |  | GO:0016600 | Cell. Component | flotillin complex |
|  |  |  |  | GO:0005198 | Mol. Function | structural molecule activity |
| BB170025B10B11 | CG3981 | FBgn0040395 | Unc-76 | GO:0008088 | Bio. Process | axon cargo transport |
|  |  |  |  | GO:0006911 | Bio. Process | phagocytosis, engulfment |
|  |  |  |  | GO:0019894 | Mol. Function | kinesin binding |
| BB170026A20C03 | CG9176 | FBgn0029090 | cngl CNG channel-like | GO:0005221 | Mol. Function | intracellular cyclic nucleotide activated cation channel activity |
| BB170026B10C12 | CG4139 | FBgn0030334 | Karl | GO:0008150 | Bio. Process | biological_process |
|  |  |  |  | GO:0005575 | Cell. Component | cellular_component |
| BB170026B10H08 | CG9045 | FBgn0002914 | Myb oncogene-like | GO:0007049 | Bio. Process | cell cycle |
|  |  |  |  | GO:0008283 | Bio. Process | cell proliferation |
|  |  |  |  | GO:0007098 | Bio. Process | centrosome cycle |
|  |  |  |  | GO:0051297 | Bio. Process | centrosome organization and biogenesis |
|  |  |  |  | GO:0000910 | Bio. Process | cytokinesis |
|  |  |  |  | GO:0007307 | Bio. Process | eggshell chorion gene amplification |
|  |  |  |  | GO:0007067 | Bio. Process | mitosis |
|  |  |  |  | GO:0007052 | Bio. Process | mitotic spindle organization and biogenesis |
|  |  |  |  | GO:0007088 | Bio. Process | regulation of mitosis |
|  |  |  |  | GO:0045449 | Bio. Process | regulation of transcription |
|  |  |  |  | GO:0006355 | Bio. Process | regulation of transcription, DNA-dependent |
|  |  |  |  | GO:0007051 | Bio. Process | spindle organization and biogenesis |
|  |  |  |  | GO:0031523 | Cell. Component | Myb complex |
|  |  |  |  | GO:0005634 | Cell. Component | nucleus |
|  |  |  |  | GO:0003677 | Mol. Function | DNA binding |
|  |  |  |  | GO:0016563 | Mol. Function | transcription activator activity |
|  |  |  |  | GO:0003700 | Mol. Function | transcription factor activity |
| BB170027B20E07 | CG32703 | FBgn0052703 | ERK7 | GO:0006468 | Bio. Process | protein amino acid phosphorylation |
|  |  |  |  | GO:0004707 | Mol. Function | MAP kinase activity |
|  |  |  |  | GO:0004674 | Mol. Function | protein serine/threonine kinase activity |
| BB170028A10F03 | CG9769 | FBgn0037270 |  | GO:0048102 | Bio. Process | autophagic cell death |
|  |  |  |  | GO:0035071 | Bio. Process | salivary gland cell autophagic cell death |
|  |  |  |  | GO:0006413 | Bio. Process | translational initiation |
|  |  |  |  | GO:0005852 | Cell. Component | eukaryotic translation initiation factory 3 complex |
|  |  |  |  | GO:0003743 | Mol. Function | translation initiation factor activity |
| BB170028A10F04 | CG32465 | FBgn0052465 |  | GO:0006911 | Bio. Process | phagocytosis, engulfment |
|  |  |  |  | GO:0004091 | Mol. Function | carboxylesterase activity |
|  |  |  |  | GO:0042043 | Mol. Function | neurexin binding |
| BB170028A10F04 | CG34127 | FBgn0083963 |  | GO:0006911 | Bio. Process | phagocytosis, engulfment |
|  |  |  |  | GO:0004091 | Mol. Function | carboxylesterase activity |
|  |  |  |  | GO:0042043 | Mol. Function | neurexin binding |
| BB170030A10F07 | CG3004 | FBgn0030142 |  | GO:0005834 | Cell. Component | heterotrimeric G-protein complex |
|  |  |  |  | GO:0003924 | Mol. Function | GTPase activity |
| BB170030A20E12 | CG6860 | FBgn0032633 |  | GO:0007265 | Bio. Process | Ras protein signal transduction |
| BB170030B10F12 | CG5214 | FBgn0037891 |  | GO:0006099 | Bio. Process | tricarboxylic acid cycle |
|  |  |  |  | GO:0005811 | Cell. Component | lipid particle |
|  |  |  |  | GO:0009353 | Cell. Component | mitochondrial oxoglutarate dehydrogenase complex |
|  |  |  |  | GO:0004149 | Mol. Function | dihydrolipoyllysine-residue succinyltransferase activity |
| BB170031A10B11 | CG9849 | FBgn0034803 |  | GO:0007521 | Bio. Process | muscle cell fate determination |
|  |  |  |  | GO:0005576 | Cell. Component | extracellular region |
| BB170031A20H10 | CG11488 | FBgn0031231 |  | GO:0006412 | Bio. Process | translation |
|  |  |  |  | GO:0005762 | Cell. Component | mitochondrial large ribosomal subunit |
|  |  |  |  | GO:0030529 | Cell. Component | ribonucleoprotein |
|  |  |  |  | GO:0003735 | Mol. Function | structural constituent of ribosome |
| BB170031A20H10 | CG5055 | FBgn0000163 | baz bazooka | GO:0045176 | Bio. Process | apical protein localization |
|  |  |  |  | GO:0008356 | Bio. Process | asymmetric cell division |
|  |  |  |  | GO:0055059 | Bio. Process | asymmetric neuroblast division |
|  |  |  |  | GO:0008105 | Bio. Process | asymmetyric protein localization |
|  |  |  |  | GO:0045167 | Bio. Process | asymmetric protein localization during cell fate commitment |
|  |  |  |  | GO:0045175 | Bio. Process | basal protein localization |
|  |  |  |  | GO:0007298 | Bio. Process | border follicle cell migration |
|  |  |  |  | GO:0007043 | Bio. Process | cell-cell junction assembly |
|  |  |  |  | GO:0000910 | Bio. Process | cytokinesis |
|  |  |  |  | GO:0007163 | Bio. Process | establishment and/or maintenance of cell polarity |
|  |  |  |  | GO:0045197 | Bio. Process | establishment and/or maintenance of epithelial cell apical/basal polarity |
|  |  |  |  | GO:0045196 | Bio. Process | establisment and/or maintenance of neuroblast polarity |
|  |  |  |  | GO:0016332 | Bio. Process | establishment and/or maintenance of polarity of embryonic epithelium |
|  |  |  |  | GO:0035089 | Bio. Process | establishment of apical/basal cell polarity |
|  |  |  |  | GO:0040001 | Bio. Process | establishment of mitotic spindle localization |
|  |  |  |  | GO:0007377 | Bio. Process | germ-band extension |
|  |  |  |  | GO:0007294 | Bio. Process | germarium-derived oocyte fate determination |
|  |  |  |  | GO:0001738 | Bio. Process | morphogenesis of a polarized epithelium |
|  |  |  |  | GO:0002009 | Bio. Process | morphogenesis of an epithelium |
|  |  |  |  | GO:0007309 | Bio. Process | oocyte axis determination |
|  |  |  |  | GO:0007299 | Bio. Process | ovarian follicle cell adhesion |
|  |  |  |  | GO:0008104 | Bio. Process | protein localization |
|  |  |  |  | GO:0007416 | Bio. Process | synaptogenesis |
|  |  |  |  | GO:0045186 | Bio. Process | zonula adherens assembly |
|  |  |  |  | GO:0005912 | Cell. Component | adherens junction |
|  |  |  |  | GO:0045179 | Cell. Component | apical cortex |
|  |  |  |  | GO:0043296 | Cell. Component | apical junction complex |
|  |  |  |  | GO:0045177 | Cell. Component | apical part of cell |
|  |  |  |  | GO:0016324 | Cell. Component | apical plasma membrane |
|  |  |  |  | GO:0016327 | Cell. Component | apicolateral plasma membrane |
|  |  |  |  | GO:0005938 | Cell. Component | cell cortex |
|  |  |  |  | GO:0005913 | Cell. Component | cell-cell adherens junction |
|  |  |  |  | GO:0005737 | Cell. Component | cytoplasm |
|  |  |  |  | GO:0005914 | Cell. Component | spot adherens junction |
|  |  |  |  | GO:0035003 | Cell. Component | subapical complex |
|  |  |  |  | GO:0005515 | Mol. Function | protein binding |
|  |  |  |  | GO:0005080 | Mol. Function | protein kinase C binding |
| BB170032A10B07 | CG16779 | FBgn0037698 |  |  |  |  |
| twsxxxxxxxxxxx | CG6235 | FBgn0004889 | tws twins | GO:0001700 | Bio. Process | embryonic development via the syncytial blastoderm |
|  |  |  |  | GO:0007447 | Bio. Process | imaginal disc pattern formation |
|  |  |  |  | GO:0000090 | Bio. Process | mitotic anaphase |
|  |  |  |  | GO:0006470 | Bio. Process | protein amino acid dephosphorylation |
|  |  |  |  | GO:0007088 | Bio. Process | regulation of mitosis |
|  |  |  |  | GO:0007423 | Bio. Process | sensory organ development |
|  |  |  |  | GO:0016055 | Bio. Process | Wnt receptor signaling pathway |
|  |  |  |  | GO:0005737 | Cell. Component | cytoplasm |
|  |  |  |  | GO:0000159 | Cell. Component | protein phosphatase type 2A complex |
|  |  |  |  | GO:0008601 | Mol. Function | protein phosphatase type 2A regulator activity |
|  |  |  |  | GO:0004722 | Mol. Function | protein serine/threonine phosphatase activity |
