# Supplementary material for: Meta-analysis of genome-wide expression patterns associated with behavioral maturation in honey bees
Source: BMC Genomics. 2008 Oct 24;9:503. doi: 10.1186/1471-2164-9-503 (PMC2582039; doi:10.1186/1471-2164-9-503)
Supplement: Additional file 4 — Gene Ontology information for transcripts identified in four or more studies. Gene Ontology (molecular function or mol. function, biological process or bio. process, and cellular component or cell. component) information for 12 transcripts with significant differential expression in four or more studies. [file 1471-2164-9-503-S4.doc]

## Additional file 4

**Gene Ontology (molecular function or mol. function, biological process or bio. process, and cellular component or cell. component) information for 12 transcripts with significant differential expression in four or more studies.**

| ***Transcript*** | ***Computed Gene (CG) Symbol*** | ***FlyBase ID*** | ***Gene Name*** | ***GO*** | ***GO Category*** | ***GO Description*** |
| --- | --- | --- | --- | --- | --- | --- |
| BB170024B20H07 | CG6186 | FBgn0022355 | Tsf1 Transferrin 1 | GO:0006879 | Bio. Process | cellular iron ion homeostasis |
|  |  |  |  | GO:0006952 | Bio. Process | defense response |
|  |  |  |  | GO:0006826 | Bio. Process | iron ion transport |
|  |  |  |  | GO:0008199 | Mol. Function | ferric iron binding |
|  |  |  |  | GO:0005381 | Mol. Function | iron ion transmembrane transporter activity |
| BB160004B20A10 | CG17737 | FBgn0035423 |  | GO:0006412 | Bio. Process | translation |
| BB170020B10D07 | CG9307 | FBgn0038180 | Cht5 | GO:0004568 | Mol. Function | chitinase activity |
| BB170013A20H11 | CG31997 | FBgn0051997 |  | GO:0008150 | Bio. Process | unknown |
|  |  |  |  | GO:0005575 | Cell. Component | unknown |
|  |  |  |  | GO:0003674 | Mol. Function | unknown |
| BB170012B20G09 | CG7470 | FBgn0037146 |  | GO:0006561 | Bio. Process | proline biosynthetic process |
|  |  |  |  | GO:0005811 | Cell. Component | lipid particle |
|  |  |  |  | GO:0005743 | Cell. Component | mitochondrial inner membrane |
|  |  |  |  | GO:0005739 | Cell. Component | mitochondrion |
|  |  |  |  | GO:0017084 | Mol. Function | delta 1-pyrroline-5-carboxylate synthetase activity |
|  |  |  |  | GO:0004349 | Mol. Function | glutamate 5-kinase activity |
|  |  |  |  | GO:0004350 | Mol. Function | glutamate-5-semialdehyde dehydrogenase activity |
| BB160024A10G05 | CG5220 | FBgn0038471 |  | GO:0008650 | Mol. Function | rRNA (uridine-2'-O-)-methyltransferase activity |
| BB170003A10E02 | CG5840 | FBgn0038516 |  | GO:0004735 | Mol. Function | pyrroline-5-carboxylate reductase activity |
| BB160009A20G09 | CG30035 | FBgn0050035 |  | GO:0008643 | Bio. Process | carbohydrate transport |
|  |  |  |  | GO:0016021 | Cell. Component | integral to membrane |
|  |  |  |  | GO:0005355 | Mol. Function | glucose transmembrane transporter activity |
| BB170024B10C11 | CG10662 | FBgn0032832 | sick sickie | GO:0050829 | Bio. Process | defense response to Gram-negative bacterium |
|  |  |  |  | GO:0005575 | Cell. Component | ND |
| BB160007A10B09 | CG17762 | FBgn0030412 | tomosyn | GO:0007163 | Bio. Process | establishment and/or maintenance of cell polarity |
|  |  |  |  | GO:0007269 | Bio. Process | neurotransmitter secretion |
|  |  |  |  | GO:0016079 | Bio. Process | synaptic vesicle exocytosis |
|  |  |  |  | GO:0016082 | Bio. Process | synaptic vesicle priming |
|  |  |  |  | GO:0008021 | Cell. Component | synaptic vesicle |
|  |  |  |  | GO:0017075 | Mol. Function | syntaxin-1 binding |
| BB160024B10F06 | CG9907 | FBgn0003036 | para paralytic | GO:0045433 | Bio. Process | male courtship behavior, veined wing generated song production |
|  |  |  |  | GO:0046680 | Bio. Process | response to DDT |
|  |  |  |  | GO:0046684 | Bio. Process | response to pyrethroid |
|  |  |  |  | GO:0006814 | Bio. Process | sodium ion transport |
|  |  |  |  | GO:0005887 | Cell. Component | integral to plasma membrane |
|  |  |  |  | GO:0005886 | Cell. Component | plasma membrane |
|  |  |  |  | GO:0001518 | Cell. Component | voltage-gated sodium channel complex |
|  |  |  |  | GO:0005248 | Mol. Function | voltage-gated sodium channel activity |
| BB170010A10A08 | CG31634 | FBgn0051634 | Oatp26F | GO:0015711 | Bio. Process | organic anion transport |
|  |  |  |  | GO:0008514 | Mol. Function | organic anion transmembrane transporter activity |
